# Comparison of REST Cistromes across Human Cell Types Reveals Common and Context-Specific Functions

**DOI:** 10.1371/journal.pcbi.1003671

**Published:** 2014-06-12

**Authors:** Shira Rockowitz, Wen-Hui Lien, Erika Pedrosa, Gang Wei, Mingyan Lin, Keji Zhao, Herbert M. Lachman, Elaine Fuchs, Deyou Zheng

**Affiliations:** 1 Department of Genetics, Albert Einstein College of Medicine, Bronx, New York, New York, United States of America; 2 Howard Hughes Medical Institute, Laboratory of Mammalian Cell Biology & Development, The Rockefeller University, New York, New York, United States of America; 3 Department of Psychiatry and Behavioral Sciences, Albert Einstein College of Medicine, Bronx, New York, New York, United States of America; 4 Systems Biology Center, National Heart, Lung, and Blood Institute, National Institute of Health, Bethesda, Maryland, United States of America; 5 Department of Neuroscience, Albert Einstein College of Medicine, Bronx, New York, New York, United States of America; 6 The Saul R. Korey Department of Neurology, Albert Einstein College of Medicine, Bronx, New York, New York, United States of America; Rutgers University, United States of America

## Abstract

Recent studies have shown that the transcriptional functions of REST are much broader than repressing neuronal genes in non-neuronal systems. Whether REST occupies similar chromatin regions in different cell types and how it interacts with other transcriptional regulators to execute its functions in a context-dependent manner has not been adequately investigated. We have applied ChIP-seq analysis to identify the REST cistrome in human CD4+ T cells and compared it with published data from 15 other cell types. We found that REST cistromes were distinct among cell types, with REST binding to several tumor suppressors specifically in cancer cells, whereas 7% of the REST peaks in non-neuronal cells were ubiquitously called and <25% were identified for ≥5 cell types. Nevertheless, using a quantitative metric directly comparing raw ChIP-seq signals, we found the majority (∼80%) was shared by ≥2 cell types. Integration with RNA-seq data showed that REST binding was generally correlated with low gene expression. Close examination revealed that multiple contexts were correlated with reduced expression of REST targets, e.g., the presence of a cognate RE1 motif and cellular specificity of REST binding. These contexts were shown to play a role in differential corepressor recruitment. Furthermore, transcriptional outcome was highly influenced by REST cofactors, e.g., SIN3 and EZH2 co-occupancy marked higher and lower expression of REST targets, respectively. Unexpectedly, the REST cistrome in differentiated neurons exhibited unique features not observed in non-neuronal cells, e.g., the lack of RE1 motifs and an association with active gene expression. Finally, our analysis demonstrated how REST could differentially regulate a transcription network constituted of miRNAs, REST complex and neuronal factors. Overall, our findings of contexts playing critical roles in REST occupancy and regulatory outcome provide insights into the molecular interactions underlying REST's diverse functions, and point to novel roles of REST in differentiated neurons.

## Author Summary

The RE-1 silencing transcription factor (REST) binds to DNA and has been shown to repress neuronal genes in non-neuronal systems, but more recent studies have expanded its functions much beyond this. At the molecular level, REST acts cooperatively with other proteins to execute its transcriptional regulatory roles. The dynamics of REST binding and cofactor recruitment and its association with the underlying DNA sequence remain unclear. Here, we have applied chromatin immunoprecipitation and deep sequencing to identify REST binding across 16 different cell types, including neurons. Our results demonstrate that REST binding events are dynamic and quite distinct among cells and that REST binding is generally associated with low gene expression. Closer examination finds that the context of the DNA sequence at REST bound sites is associated with the lower expression of REST-associated targets and that different contexts correlate with different cofactor recruitment. These in turn have an effect on the expression of REST targets. REST targets in human neurons, however, are drastically different from those in other cell types. These findings provide insights into the effect of genomic and cellular contexts on REST's diverse functions and point to distinct and novel roles for REST in neurons.

## Introduction

The *REST* (RE1-silencing transcription factor) [1], also known as *NRSF* (Neural Restrictive Silencing Factor) [2] and *XBR* (X2 Box Repressor) [3], encodes a zinc-finger transcription factor that was initially shown to repress neuronal genes in non-neuronal tissues and neural progenitors. It has since been shown to play a broad range of roles in neuronal differentiation and development [4]–[6], such as fine-tuning neural gene expression [7] and modulating synaptic plasticity [8]. REST is necessary for the maintenance of self-renewal capacity of neural stem cells (NSCs), as its knockdown led to a lower mitotic index and a higher rate of early neuronal differentiation [5]. REST has also been implicated as a tumor suppressor in breast cancer, colorectal cancer and small cell lung cancer, and as an oncogene in neuroblastomas, medulloblastomas and pheochromocytomas, which are associated with von Hippel-Lindau syndrome [9], [10]. These findings show that REST plays diverse roles in multiple cellular processes.

In addition to the 21-bp DNA sequence bound by REST (termed the RE1 motif), an array of cofactors have been found to interact and cooperate with REST, including SIN3, CoREST, Polycomb Repressive Complexes (PRCs), and various histone deacetylases (HDACs) [9], [11], [12]. Many of these cofactors are chromatin modifiers or are associated with enzymes that have effects on post-translational histone modifications, suggesting that at the molecular level REST functions as a platform for the recruitment of multiple chromatin modifiers and that together they orchestrate gene regulation [9], [11], [12]. In fact, REST occupancy has been found to correlate with an increase of repressive and a decrease of active histone modifications [13]. Not all of the REST cofactors, however, are recruited to each of the REST-bound loci concomitantly. For example, a study of REST occupancy in eight RE1 loci in mouse NSCs found the existence of four distinct configurations of REST and its cofactors: REST-Sin3b-CoREST-HDAC1/2, REST- Sin3b-CoREST, REST-CoREST, and REST-Sin3b-HDAC1/2 [14]. An independent genome-wide study showed that approximately half of the REST-bound sites in mouse ESCs were associated with REST cofactors (in various combinations of the Sin3 and CoREST family members) and that genes targeted by REST together with its cofactors showed more repression than genes bound by REST alone [15]. These studies indicate that differential recruitment of REST cofactors can potentially orchestrate distinct transcriptional outcomes. Moreover, REST acts as a repressor at only a subset of RE1 containing genes [16], [17]; in particular, REST and its splicing variants have been reported to activate a variety of genes in certain cell types and conditions, such as *CHRNB2*, *CRH*, *PAX4*, and *OPRM1* [18]–[23].

Previous genome-wide studies have characterized REST targets in human Jurkat cells [24], K562 cells [25], APL blasts [26], mouse embryonic [15], [27]–[29] and neural stem cells [27], [28]. Widespread switches in REST targeting during mouse neuronal and glial differentiation have also been reported [7], [30], [31]. Moreover, analysis of 1% of the human genome (the ENCODE pilot regions [32]) using ChIP-chip technology has found interesting context-dependent REST functions among cell types [33]. While these previous studies have provided important information and principles about REST chromatin interaction and gene regulation, they have not systematically addressed how REST cistromes differ across diverse human cell types. In our current study we set out to address this by integrated analysis of ChIP-seq and RNA-seq data, including primary cells and differentiated neurons. By a comprehensive analysis of ChIP-seq data for 16 cell types, including one collected for this study for CD4+ T cells and 15 cell types from the ENCODE project [34], we have identified a total of 21,134 non-redundant REST binding sites (i.e., peaks) in the human genome. Among the REST sites in 15 “non-neuronal” cell types, only 7% were common to all. We then studied how RE1 motif status, genic location and the cellular context were related to REST binding and gene expression, as well as how these contexts were related to co-factor colocalization. Finally we compared REST occupancy between neurons and non-neuronal cells and found that REST bound to a distinct set of targets in neurons. Moreover, in contrast to other cell types, REST binding was largely localized to genes that were activated in differentiated neurons, as indicated by high levels of gene expression and active histone modifications. Our study provides valuable insights into the dynamic landscape of REST-chromatin interactions in the human genome and the importance of genomic and cellular contexts in modulating the outcome of REST regulation.

## Results

### REST occupancy in differentiated human cells

**Figure 1:**
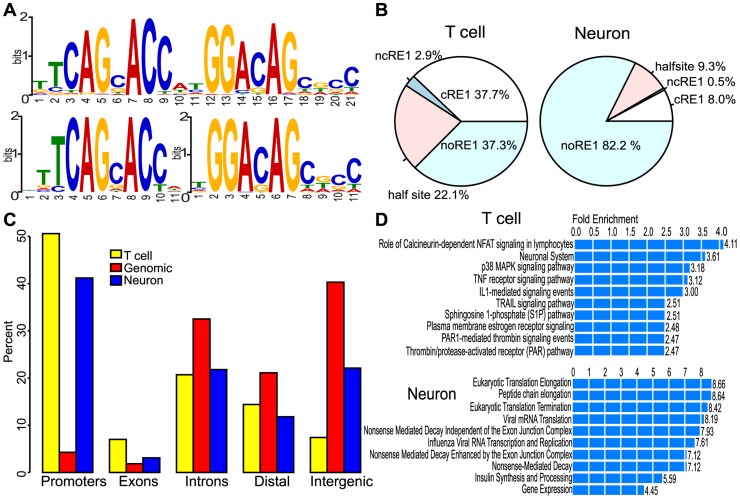
REST binding in H1-neurons and T cells. (A) Top motifs identified in T cell ChIP-seq peaks by MEME: (top) cRE1 and (bottom) left and right half-sites of the ncRE1. (B) Distribution of peaks with RE1 motifs. (C) Genomic distribution of REST peaks. (D) Pathways enriched in the REST-bound genes in T cells and neurons, from GREAT [39]; data from “Pathway Commons” were shown and presented as fold enrichment (all p-values≤1.35E-15).

To investigate the roles of REST occupancy in cell types relevant to normal physiology, we have performed a ChIP-seq analysis on human CD4+ primary T cells and compared the results with REST ChIP-seq data for 28-day-old differentiated human neurons (derived from the H1-ESC line), which were obtained from the ENCODE project [34] (Table S1). In order to make the data uniform and comparable across cell types, we called REST binding sites (i.e., ChIP-seq peaks) by the same pipeline and with consistent parameters across datasets: using the program SPP [35] and the IDR methodology recommended by the ENCODE project [34] (see Methods). As a result, we called 4,404 REST binding sites for the T cells and 5,387 for the neurons (Table 1). From the T cell REST peaks, we selected 10 sites and confirmed the binding of nine by ChIP-qPCR; the qChIP enrichments were consistent with the peak enrichment scores provided by SPP (Fig. S1). 38% of the T cell REST peaks had the canonical 21 bp RE1 motif (cRE1) (Fig. 1A), while an additional 3% contained a non-canonical RE1 motif (ncRE1), in which two halves of the cRE1 motif were separated by a 1–10 nucleotide insertion [24], [27] (Fig. 1A, bottom). Consistent with previous reports, we also found that a large fraction (22%) of the REST peaks had only one of the two half-sites. In total, 63% of REST peaks in T cells had the cRE1 motif or one of its variants. For the neuron peaks, motif analysis revealed an unexpected and different picture. Less than 10% of the REST peaks in neurons contained either the cRE1 or ncRE1 motifs (Fig. 1B). Even the enrichment of RE1 motifs in the top 600 neuronal REST peaks was marginal, occurring in 22 sites (p = 4.9E-64 from MEME [36]). A second enriched motif, GGAAA/TA, was detected among these peaks (n = 180, p = 1.6E-50) (Fig. S2). It is similar to the DNA motifs recognized by transcription factors NFATC2, dl_2 and EDS1, and it was found in 12% of the total H1-derived neuron REST peaks, compared to 3% for the T cell REST peaks and 9% of randomly selected genomic sequences.

**Table 1:** Numbers of REST peaks and REST-bound genes in each cell type.

Cell Type	Total Peaks	Cell-Specific Peaks	% of Cell Specific Peaks	Total Genes Bound	Genes with Cell-specific Peaks	% of Genes with Cell specific Peaks
A549	4,651	577	12.4%	3,258	270	8.3%
CD4+ T cell	4,404	1,118	25.4%	3,307	782	23.6%
ECC-1	5,189	34	0.7%	3,172	15	0.5%
GM12878	2,535	53	2.1%	1,733	27	1.6%
H1 ESCs	8,199	158	1.9%	4,509	43	1.0%
H1-derived neurons	5,387	3,313	61.5%	3,389	1,633	48.2%
HCT-116	2,816	16	0.6%	1,761	9	0.5%
HeLa S3	4,112	86	2.1%	2,632	53	2.0%
Hep G2	2,953	84	2.8%	1,943	50	2.6%
HL-60	4,175	3	0.1%	2,865	1	0.0%
K562	5,591	4	0.1%	3,718	2	0.1%
MCF-7	3,935	212	5.4%	2,434	117	4.8%
PANC-1	3,251	29	0.9%	2,166	23	1.1%
PFSK-1	3,513	104	3.0%	2,295	49	2.1%
SK-N-SH	4,069	157	3.9%	2,806	75	2.7%
U87	2,408	55	2.3%	1,682	36	2.1%

A comparison of the REST-bound genes further demonstrated the distinction between REST targeting in T cells and neurons. The 4,404 peaks identified in T cells were associated with 3,307 Refseq [37] genes and miRNAs [38], while the 5,387 peaks in H1-derived neurons were associate with 3,389 genes/miRNAs. Despite a previous report that RE1 motifs were prominently distributed in introns [24], 51% and 41% of the REST peaks were localized to promoter regions (−5 kb to +1 kb from transcription start sites, TSSs) in T cells and neurons, respectively. Other than promoter regions, 28% and 25% of the REST peaks were found within intragenic regions and another 14% and 12% were within 50 kb of genes (Fig. 1C). In total, 93% and 78% of the T-cell and neuronal peaks were assigned to one or more genes, respectively. Furthermore, functional analysis of the REST-bound genes using GREAT [39] revealed a dramatic disparity in the enriched pathways between these two cell types. One of the pathways defined by Pathway Commons [40] that was enriched in T cells showed clear involvement in neuronal function: neuronal system (p = 1.9E-5) (Fig. 1D), the other top enriched pathways, however, were related to functions important for lymphocytic cells. We found different categories of REST targets in neurons; the top four enriched pathways were involved in general gene expression (p = 1.1E-38) (Fig. 1D). The primary known REST function is to repress neuronal genes in non-neuronal cells [41]–[43], our data agreed with this, as this function was only found to be enriched in the T-cell REST targets. Only 487 (11%) of the T cell peaks overlapped with those from neurons, and the majority (n = 259; 53%) of these contained either a cRE1 or ncRE1 motif. Notably, 655 (73%) of the 894 genes targeted in both cell types had at least one peak that was detected in only one of the two cell types. Those common REST targets were enriched for neuronal functions, so were the T cell only REST targets, but not the neuron-only REST targets. These results suggest that REST cistromes of non-neuronal and neuronal systems may share limited overlap.

### Comparison of REST occupancy across non-neuronal cells

To extend our observation of distinct REST occupancy between neurons and non-neuronal cells, and also to gain insight into the dynamics of REST cistromes across human cell types, we decided to explore more publicly available data from the ENCODE project [34] and expand our comparison to include fourteen additional human cell lines: alveolar adenocarcinoma cells (A549), endometrial carcinoma cells (ECC1) lymphoblastoid cells (GM12878), embryonic stem cells (H1), colon carcinoma cells (HCT-116), cervical adenocarcinoma cells (HeLa S3), liver hepatocellular carcinoma cells (Hep G2), promyelocytic leukemia cells (HL-60), erythroleukemia cells (K562), breast adenocarcinoma cells (MCF-7), pancreatic carcinoma cells (PANC-1), primitive neuroectodermal tumor cells (PFSK-1), neuroblastoma cells (SK-N-SH), and glioblastoma cells (U87) (Table 1 and Table S1). These cell types represent a number of lineages. The SK-N-SH cell line is particularly interesting as it allows us to perform a comparison between normal neurons and tumorgenetic neuroblastoma cells, which have been reported to be associated with increased REST expression [44]. The resulting peak numbers from our ChIP-seq analysis pipeline are shown in Table 1, and range from 2,048 (in U87) to 8,199 (in H1 ESCs). (See Table S2 for list of peaks).

**Figure 2:**
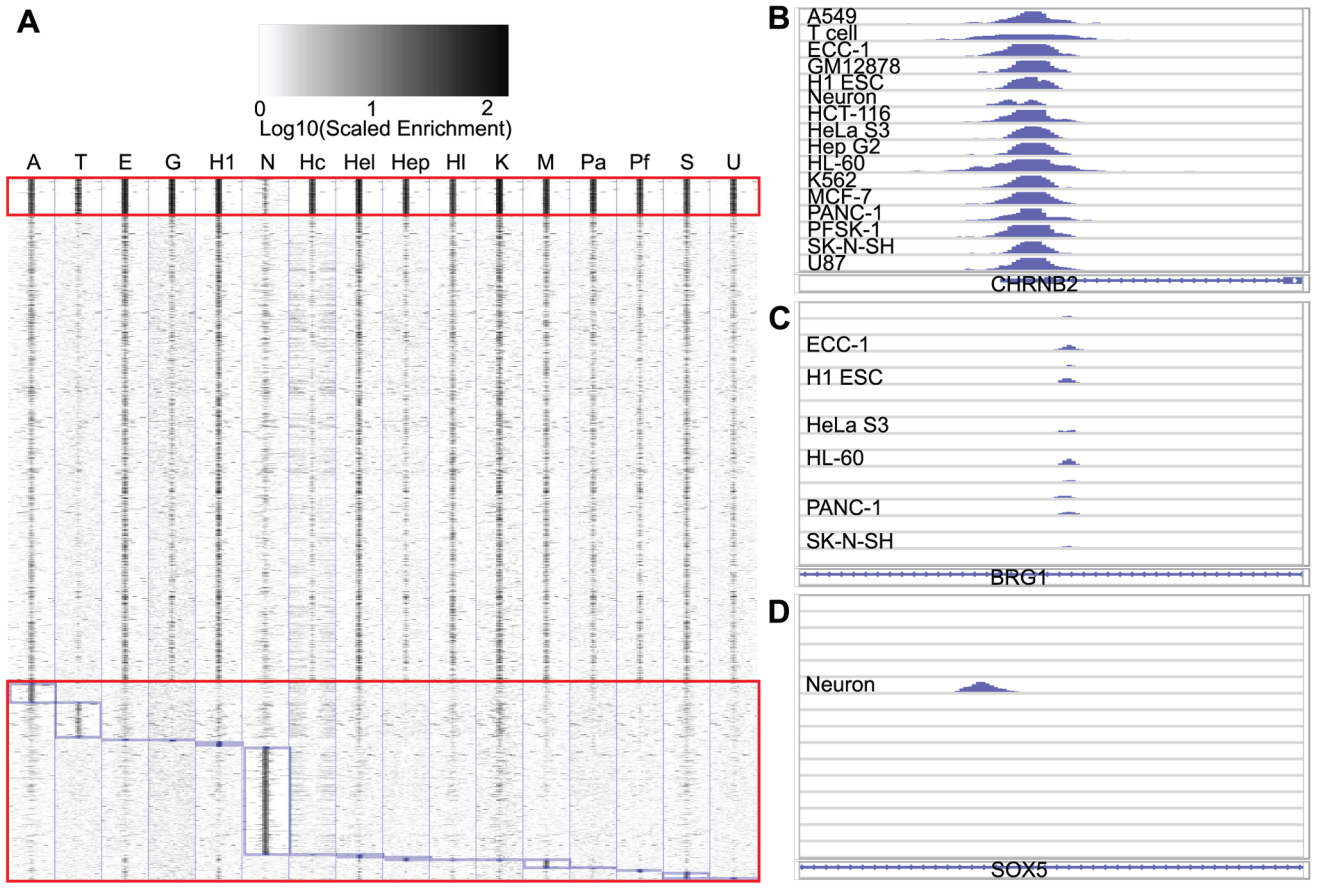
Heatmap of ChIP-seq read density across REST peaks. (A) Heatmap of maximal read coverage in 300 bp bins from −3.15 kb to +3.15 kb of the peak summits at all peaks identified from all 16 cell types (n = 21,134). The common peaks (top; n = 1,116), and cell-specific peaks (bottom; n = 6,003) are delineated from the remaining (middle; shared) peaks by red boxes and furthermore by blue boxes to demarcate cell-specific peaks for individual cell types. The labels for cell types are, A-A549, T-Tcell, E-ECC-1, G-GM12878, H1-H1 ESC, N-Neuron, Hc-HCT-116, Hel-HeLa S3, Hep-Hep G2, Hl-HL-60, K-K562, Pa-PANC-1, Pf-PFSK-1, S-SK-N-SH, U-U87. (B–D) Pileup of REST ChIP-seq reads mapped to three selected genes with common (B), shared (C), and cell-specific (D) peaks, as displayed in the IGV browser. In B–D, labels were used to mark cell types contained the peaks, as called by the SPP software.

We next evaluated REST binding sites for cell specificity. Based on the above described difference in REST binding sites between T cells and neurons and the expectation of REST repression of neuronal genes in non-neuronal cells, we decided to compare REST cistromes across all 15 non-neuronal cells first, and then brought in neurons for a final comparison. Noted that we considered those tumor cell lines derived from neural tissues (e.g., SK-N-SH) as “non-neuronal” in this report. After overlapping peaks were merged, we obtained a set of 16,913 non-redundant REST binding regions from the total of 61,801 peaks in the 15 non-neuronal cell types. Analysis of the ChIP-seq signals showed different levels of REST enrichment across these peaks among the 15 cell types (Fig. 2A), and to our surprise, only 1,116 (7%) of the merged REST peaks were consistently called by SPP in all these cell types (referred to as “common” peaks). Nevertheless, 7% is much more than expected by chance, since we obtained 0 in common when we randomly picked genomic regions, with total number and size distribution matching to those of the REST peaks in individual cell lines, and performed the same merging procedure. A similar small fraction of REST peaks were found to be common in a previous analysis of REST bindings in 1% of the human genome [33]. Interestingly, these common peaks indeed exhibited the greatest enrichment of REST ChIP-seq signals in all cell types (Fig. 2A/B).

To study cellular specificity of REST occupancy more robustly, we compared several quantitative metrics for evaluating REST ChIP-seq signals at individual peaks for differential binding across cell types (see Supplementary Methods for more details). In brief, we analyzed the number of ChIP-seq reads at the summits of the non-redundant peaks using the program seqMiner [45] and then computed Z-scores to detect cell types with significantly more ChIP-seq reads than the rest. The results showed that ChIP-seq signals for 2,690 (16%) of those non-neuronal REST peaks were significantly stronger in one cell type than in any other cell type; these were termed “cell-specific” peaks (Fig. 2A/D and Table 1). Nevertheless, a large fraction (77%) of non-neuronal REST binding sites were shared by at least two or more cell types (referred to as “shared” peaks), as illustrated in Fig. 2A. In a comparison of the common, shared (i.e., 2–14 cell types) and cell-specific peaks, we found that common peaks were more enriched with the cRE1 motif (86%) than non-common ones (53%). The GGAAA/TA motif identified in neurons was not particularly enriched in any of the other cell types, as it occurred in 5.4% of the combined non-neuronal REST-binding sites (binomial test, p = 1.7E-133). Although 78% (n = 4,240) of the neuron peaks were called only for this cell type by SPP, 22% of them exhibited significant ChIP-seq signals in other cell types as well, thus resulting in 62% of neuronal REST peaks specific to neurons by our definition. This change indicates that comparison of transcription factor occupancy across samples by simple intersection of the genomic coordinates of the ChIP-seq peaks could exaggerate the true difference significantly. Interestingly, even for the neuronal REST peaks overlapping with peaks in other cell types, the peak summits were often shifted slightly to a new position in neuronal chromatin. While the average distance between the summits of overlapping peaks found in pairs of non-neuronal cells ranged from 6 bp (GM12878 vs. Hep G2, MCF-7 vs. HL-60, and MCF-7 vs. K562) to 24 bp (A549 vs. T cell), the mean distance of REST peak summits between neurons and other cell types ranged from 26 bp (vs HCT-116) to 79 bp (vs T cell) (Fig. S3). This observation again reveals the distinction of REST occupancy in neuronal cells.

To address if chromatin factors may contribute to cell-specific REST binding, we analyzed available DNase-seq data from the ENCODE project and related them to REST binding in A549, GM12878, Hep G2, H1 ES, K562, MCF-7 and T cells. We had expected that chromatin regions bound by REST would have greater DNAse-seq signals than “potential” but unbound REST candidate sites (i.e, REST-bound in other cell lines). While this was true in four of the cell types (A549, K562, MCF7 and T cells), with REST-bound regions showing 1.5–3.0× more overlapping with DNAse-seq peaks, no difference was observed for GM12878, Hep G2 and H1 cell lines. Nevertheless, we found that DNase-seq signals (measured by read densities) at the sites with stronger REST occupancy were generally higher than sites with weaker REST occupancy in all of these seven cells (data not shown). On the other hand, DNA-seq signals at many unbound REST candidate sites still showed much greater DNase-seq read enrichment in comparison to adjacent genomic regions. Taken together, these results indicate that chromatin accessibility is not the critical factor determining REST occupancy and thus the dynamics of REST cistromes. This observation is consistent with previous finding that neither DNase hypersensitivity nor chromatin features were a good predictor of REST binding [46].

### Functional analysis of the REST targets

Next, we compared the genes and miRNAs [37], [38] that were bound and thus potentially regulated by REST. Similar to REST peaks, the numbers of REST targets varied from one cell type to another (Table 1), with a total of 10,286 genes and miRNAs bound in at least one of the 16 cell types. There was approximately a 3-fold difference between the cell type with the highest (H1 ESCs, 4,509) and the one with the lowest (U87 cells, 1,682) number of REST targets. Five pathways: neuronal system, GPCR ligand-binding, potassium channels, transmission across chemical synapses, voltage-gated potassium channels were identified as significantly enriched in the REST targets for >10 cell types (Table S3). All of these pathways are important for neuronal function. Interestingly, pathways involved in translation (e.g., peptide chain elongation) were significantly enriched in REST targets in A549, HL-60, PFSK-1, and SK-N-SH cells, along with neuronal pathways, and in REST targets in neurons, but to the exclusion of top neuronal pathways (Table S4). Notably, REST-bound genes in these pathways were predominantly the same set of targets shared by these cell types. In addition, nearly all of the genes (n = 856) targeted by REST in all 15 non-neuronal cells contained a common REST peak (i.e., called in all cells). Among these common genes, only 1.2% (n = 10) were bound by REST at different genomic sites in any of the 15 non-neuronal cell types and 41% (n = 353) were also bound by REST in neurons. Interestingly, one of those genes targeted by REST in all cell types except neurons was REST itself, for which negative auto-regulatory feedback has been proposed [24].

REST was found to bind proximally to many of the well-characterized neuronal genes in various brain cancer cell lines: PFSK-1, SK-N-SH, U87 and in H1-derived neurons, a phenomenon previously reported [41]–[43]. We compiled a list of 15 known REST target genes from the literature, including *BDNF* [43], [47], *CALB1* [48], *L1CAM* [49], *CHAT* [50], *GRIA2* [51], *CHRM4* [52], *NRCAM* [53], *GRIN1* [54], *STMN2* [52], *SCG2* [55], *SYN1* [52], *SYP* [56], *SYT4* [48], *GLRA1* [52], *CHRNB2* [52]. All of these genes were REST-bound in 14 or more cell types. Among these, 6 of them (*GLRA1*, *GRIA2*, *SCG2*, *CALB1*, *STMN2*, *CHRNB2*) were bound in all 16 cell types, including neurons. The remaining nine genes displayed variable binding, with any lack of REST occupancy occurring in only four cell types: neurons, HCT-116 cells, PANC-1 cells, or Hep G2 cells. This result indicates that our observation of neurons as an “outlier” is unlikely due to some experimental technical biases (e.g., off target immunoprecipitation) in ChIP-seq analysis. Instead, it suggests that the prevalent view of REST having similar functions in non-neuronal systems through the repression of neuronal genes may have arisen from a systematic experimental bias that the same small set of genes has been examined repeatedly in previous studies. In addition, of the 52 genes that were upregulated upon REST knockdown in HEK293 cells [57], 54% (n = 28) of them showed variable REST binding among the 16 cells. 11 of these genes exhibited differential REST occupancy in neurons, with 10% (n = 5) of them bound by REST exclusively in neurons and 12% (n = 6) bound in other cell types, but not in neurons. This result suggests that a large fraction of genes repressed by REST are direct REST targets in non-neuronal cells.

As REST has been previously implicated in a variety of cancers, we decided to look into whether there were any cancer specific REST targets. A comparison of the REST occupancy in differentiated cell types (T cells and neurons) with the 13 cancer-derived cell lines revealed that several tumor suppressor genes (*OSMR*, *MYO1A*, *THRB*, *FRMD3*, *LOXL4*, *CEACAM3*, *TRH*) were bound by REST in all of the cancer cell lines, but in neither neurons nor T cells. Conversely, IL-7 receptor (*IL7R*) and *LAMA2*, two genes that are upregulated in a number of cancers [58], [59], were targeted by REST only in the two non-tumorigenic differentiated cell types. Notably, in H1 ESCs the REST binding patterns at all of these genes (except *LAMA2*) matched with those in the tumor cell lines, suggesting that REST regulation of these genes may have a role in cell proliferation, since active growth is a common feature of ESCs and tumor cells.

### Correlation of REST binding with low gene expression

**Figure 3:**
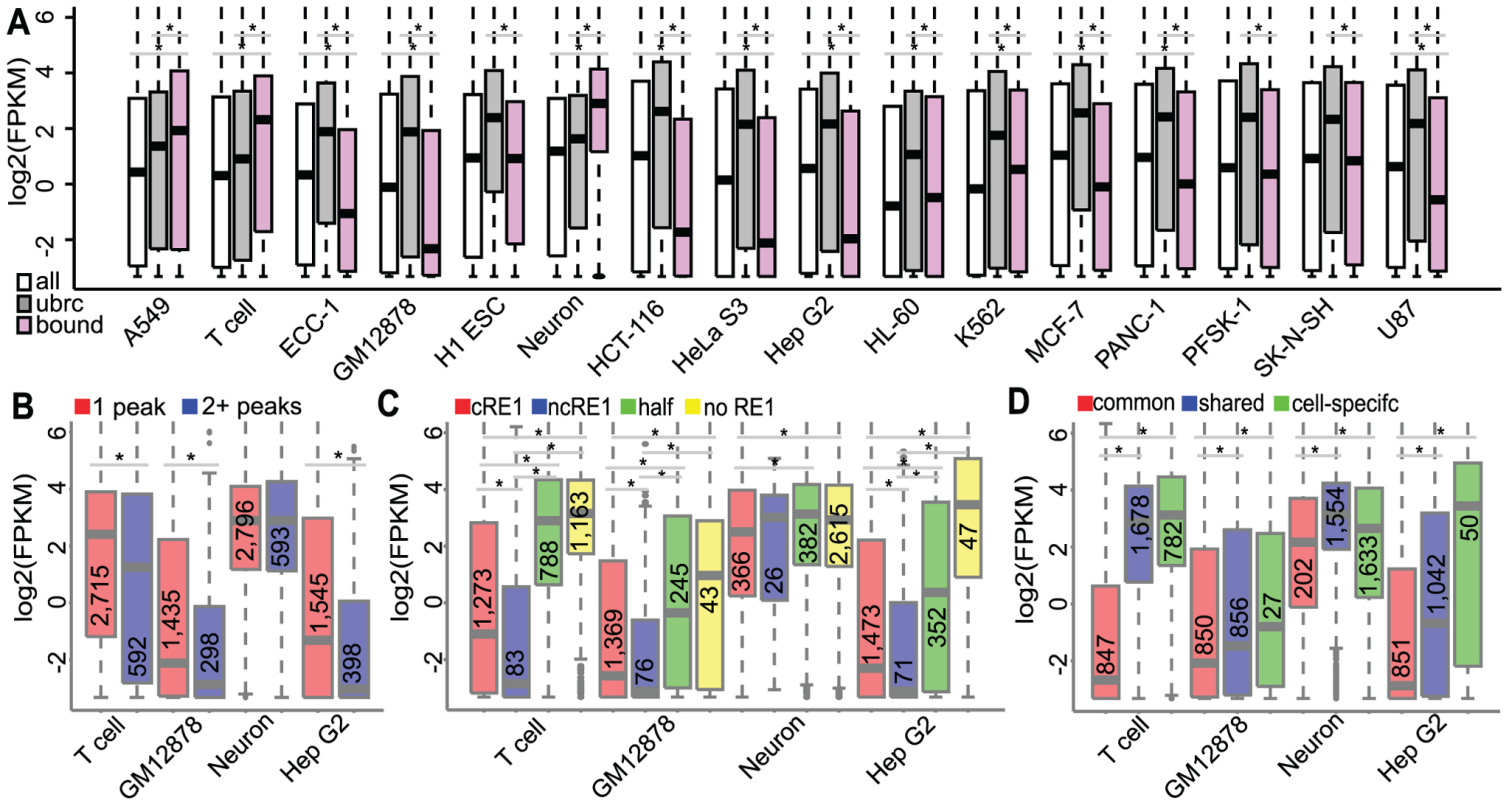
Transcription of REST targets bound by REST in different contexts. (A) Comparison of the expression of REST-bound targets (b) vs. unbound REST candidate genes (ubrc; bound in other cell types except the subject one) or all genes (all). (B–D) Expression differences of REST targets: (B) with one peak vs. more peaks; (C) bound by peaks with cRE1, ncRE1, half-site or no RE1; (D) associated to common, shared, or cell-specific peaks. Asterisks mark significant differences in expression (p<0.05). Numbers in boxes are the number of genes whose peak has the specified context. Data plotted as log2(FPKM+0.1) and shown here for T cells, GM12878 cells, neurons and Hep G2 cells; data for other cells are in Table S6.

We next sought to better understand the transcriptional effect of REST occupancy, by integrating REST cistromics data and transcriptomics data. We utilized RNA-seq data (Table S5) and the TopHat/Cufflinks software suite [60] to determine gene expression levels in all of the cell types (Fig. S4). Hierarchical clustering analysis of gene expression showed that cell types of similar lineages and functions were grouped together, affirming the quality of our RNA-seq data (Fig. S4). We took the combined REST targets (n = 10,286) in all 16 cells and then for a particular cell type we compared the transcription of the bound (b) to the unbound REST candidates (ubrc = 10,286-b; approximate for potential REST targets) as well as to the transcription of all genes. For the majority (13) of the 16 cell types, REST bound genes were transcribed at a level significantly lower (2.4–36 fold lower; p<2E-16) than the unbound REST candidates (Fig. 3A), congruent with REST's primary role as a repressor [9]. In addition, in the majority (11) of the cell types lower expression of REST-bound genes compared to all genes was observed (significant in 8; p<2E-5). In A549 cells, T cells and neurons, REST-bound targets exhibited a significantly higher expression (Fig. 3A and Table S6). The predominantly repressive function of REST was further supported by very low expression of the 15 known REST targets described above (medians of FPKMs were 0.02–1.1 in all non-neuronal cell types but 16.2 in neurons). We were not surprised to find that REST targets were highly expressed in neurons as it has been reported that REST could bind and activate neuronal genes [9]. We were, however, surprised to see that REST-associated genes also exhibited greater expression than unbound REST candidate targets in A549 cells and T cells. Out of the top 200 most expressed genes in individual cell types, 37%–59% were REST-bound in A549 cells, neurons or HL-60 cells, while <23% were bound by REST in all other cell types. Interestingly, neurons, T cells and A549 cells had high proportions of REST peaks located to promoters and a higher percentage of REST peaks without an RE1 motif than the other cell types, the significance of which needs further investigation.

Current data also allows us to make an interesting comparison between the SK-N-SH neuroblastoma cell line and neurons. It has been reported that elevated expression of *REST* is associated with neuroblastomas [44]. RNA-seq data showed that *REST* was indeed more highly expressed in the SK-N-SH line (6.3 FPKM) than neurons (2.7 FPKM). Relatively few REST peaks (n = 386) overlapped in these two cell types. We found that a variety of neuronal pathways were enriched among the genes that were expressed at a lower level in SK-N-SH and bound by REST in SK-N-SH cells but not in neurons, while many genes involved with translation and RNA processing were uniquely bound and more highly expressed in neurons. As expected, genes in neuronal system pathways had higher expression in neurons than in SK-N-SH cells (median FPKM of 5.6 vs. 3.0), but even those bound by REST only in neurons (n = 17) were highly expressed (median FPKM 34.7 vs. 0.3 FKPM for those bound in SK-N-SH cells only). Interestingly, the tumor suppressors *TRH* and *MTSS1* were not expressed in SK-N-SH cells (0 and 0.27 FPKM, respectively) but were in neurons (3.7 and 20 FPKM, respectively). In contrast, the oncogene β-catenin and proto-oncogene *IL-7* were much more highly expressed in SK-N-SH cells (99 and 4.7 FPKM, respectively) than neurons (61 and 0.4 FPKM, respectively). These data support the view that REST may have a direct functional role in cancer progression by regulating oncogenes and tumor suppressors and the point that REST does not always repress its targets.

### Roles of genomic and cellular contexts in REST-mediated gene repression

Previous studies show that REST binding does not always lead to gene repression and that in some cases it is conversely correlated with gene activation. In addition, it has been shown that the sequence bound by transcription factors can determine cofactor specificity [61]–[64] for a number of proteins such as for the transcription factor NF-κB [61], hormone activated estrogen receptors [62], and the glucocorticoid receptor [63], [64]; thus, we wondered if the context of REST binding plays a role in gene regulation.

By analyzing the REST peak numbers, we found that genes with more REST peaks generally exhibited lower levels of expression than those with a single peak. This observation persisted in all 16 of the cell types and was statistically significant in 15 (Fig. 3B shows data for GM12878, Hep G2, T cells and neurons; data for all cell types in Table S6; about 1.5–68 fold lower). This finding suggests that there may be an additive dosage effect of REST occupancy on the repression of its targets.

We also found that genes with REST peaks containing RE1 motifs (either cRE1 or ncRE1) generally exhibited lower expression levels than those without RE1 motifs or with half-sites, consistently across all cell types (Fig. 3C, Fig. S5, and Table S6; about 1.4–164 fold lower). Intriguingly, despite the low occurrence of RE1 motifs in neuron peaks, this trends held. However, even the REST bound genes with RE1 motifs had a higher expression level in neurons than in any other cell types (median of 5.6 FPKM in neurons vs. <1.3 FPKM in others, Table S6). Interestingly, genes with ncRE1-peaks tended to exhibit even lower levels of expression than those with cRE1, in agreement with a previous report [13]. This also persisted in 14 of the cell types and was statistically significant in 10 (Fig. 3C, Fig. S5, and Table S6). Notably, REST genes with RE1 motifs had lower expression than those without RE1 motifs and overall stronger peaks were associated with lower gene expression, except in neurons (data not shown).

Next, we examined whether genes consistently bound by REST in all cell types were as lowly expressed as genes that were bound variably (shared or cell-specific). Indeed, we found that genes with a common REST binding site exhibited lower levels of expression than those with shared or cell-specific sites (Fig. 3D and Table S6; about 1.7–195 fold lower). Interestingly, the highest levels of expression for these common genes occurred in neurons exclusively (median expression of 4.4 FPKM).

Finally, we compared the expression of different groups of REST targets that were separated based on the relative locations of REST binding. In 11 of the cell lines, genes bound by REST in their bodies (introns or exons) exhibited significantly lower expression levels than those with REST in their promoters (data for all cell types in Table S6; about 1.4–69 fold lower). This is quite interesting, since the effect of REST on gene expression has been mostly studied through its binding at promoter regions. On the other hand, it has been reported that REST binding within 50 bp of the TATA box in neuronal cells (but not in non-neuronal cells) was correlated with gene activation [18]. Indeed, those genes bound by REST at their promoters with peak summits located <50 bp from the TSS exhibited higher expression levels (Table S6; 1.8–5.4 fold higher) than even unbound REST candidate genes. This difference was found in 9 of the cell types, and was statistically significant in 7. This finding is probably related to the emerging view that transcription factor binding at enhancers has a greater effect on gene expression than binding at promoters, where many factors likely act competitively or coordinately.

As previously mentioned, data from A549 cells, T cells and neurons did not indicate repression of REST targets. No dosage-associated expression difference was detected for REST binding in neurons. Nevertheless the presence of RE1 motifs, cell specificity, and location of REST peaks still made a difference in terms of the extent of REST repression (Table S6). These three cell types had the largest percentages of REST peaks in the promoter regions (34.6%, 50.6%, and 41.2% for A549 cells, T cells, and neurons, respectively, in comparison with 14.2–28.1% of peaks in other cell types) and the smallest percentages of their peaks contained a cRE1 or ncRE1 motif (48.2%, 40.6%, and 8.5% A549 cells, T cells, and neurons, respectively, in comparison with 51.3%–83.8% in other cell types). Both of these two features showed a strong bias towards higher expression of the REST targets.

Since these aforementioned factors were not independent, we performed a logistic regression to determine the individual contributions of these factors to predict the transcription outcome of a REST target as expressed (FPKM≥1) or not expressed (FPKM<1). The results indicated that genes next to RE1 motif peaks, common REST peaks, and intragenic/distal peaks were 2.6, 1.9 and 1.3 times more likely to not be expressed than genes next to RE1-free peaks, non-common REST peaks, and promoter peaks, respectively, suggesting that motif status and cellular context were the two primary factors.

### Contexts are associated with differential recruitment of REST cofactors

**Figure 4:**
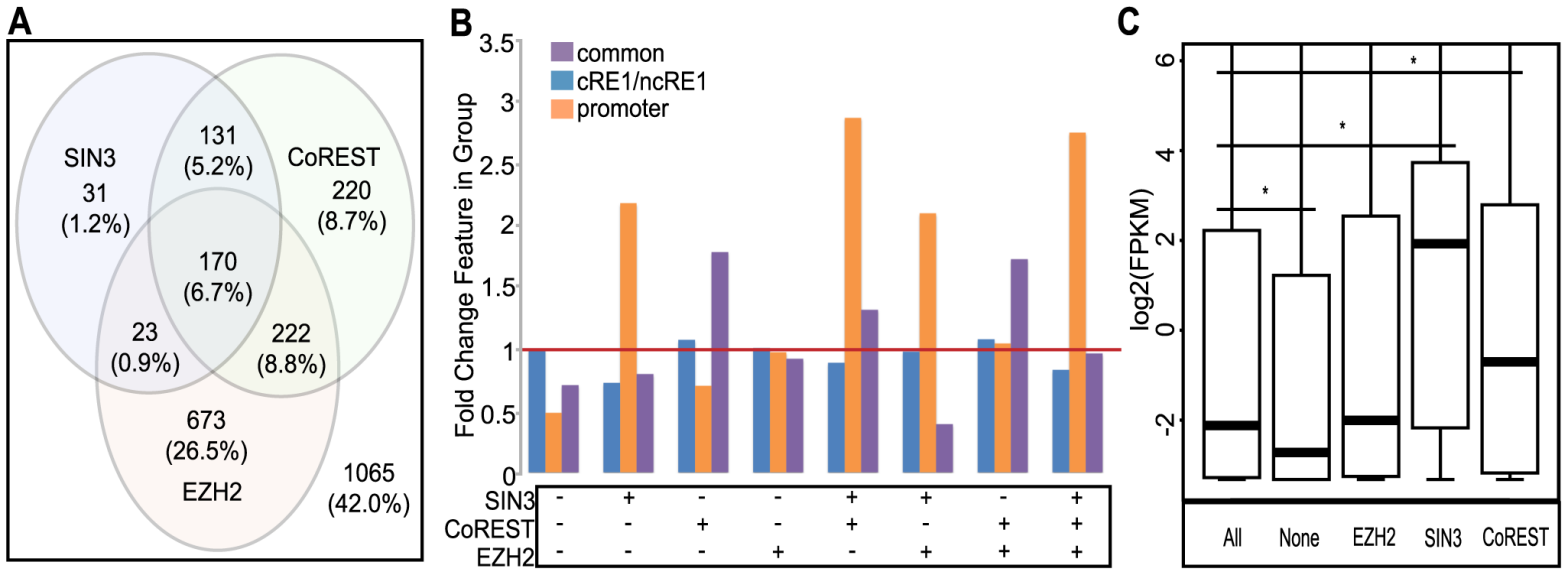
Colocalization of REST and its cofactors and their relationship with gene expression. (A) Venn diagram of colocalization of SIN3, COREST, and EZH2 at REST peaks. The square constitutes all REST peaks. (B) Context enrichment of REST peaks bound by different cofactors; fold enrichment is compared to all REST peaks. (C) Expression of REST targets with different cofactors, plotted as log2(FPKM+0.1); asterisks mark significant differences. Data shown here are for GM12878 and data for Hep G2 are in Figure S6.

The above analyses show that context plays a role in REST regulation of its targets, which brings up a question as to whether this is due to different sets of REST cofactors being recruited. To address this, we investigated the co-occupancy of REST with CoREST, SIN3, and EZH2 (a core component of PRC2) using the ENCODE project ChIP-seq data from the two cell types: GM12878 and Hep G2 [34]. There were 17,590 SIN3, 44,065 CoREST, and 64,277 EZH2 peaks in GM12878. The corresponding numbers in Hep G2 cells were 32,019 SIN3, 51,883 CoREST and 79,275 EZH2 peaks. Note that we merged the two sets of SIN3 peaks in Hep G2. First, we found that 14%, 29% and 43% of the GM12878 REST peaks overlapped with SIN3, CoREST and EZH2, respectively. Interestingly, a previous analysis also showed that RE1 motifs were highly enriched in the SIN3A-occupied genomic sites in H1ESCs [65]. Further analysis showed that only a small fraction (7%) of REST-bound regions had all three cofactors, while a large percent (42%) were not occupied by any of these cofactors at all (Fig. 4A). In addition, most (85%) of the SIN3-REST co-binding regions were localized to sites bound by CoREST, and a large fraction (36%) of the REST-EZH2 sites were co-occupied by CoREST. Next we determined the enrichment of the contexts in each of the groups with different combinations of REST and its cofactors. We found that REST-SIN3 peaks were 2.7 times more likely than expected (in relation to all REST peaks) to be in promoters, 1.7 times more likely to contain either a half RE1 motif or no RE1 motifs. It should be noted that the TSS proximity of SIN3 has been reported previously [66]. REST-CoREST peaks were 1.5 times more likely than expected to be REST-bound in all 15 cell types (i.e. common REST peaks). REST-EZH2 peaks did not show an increased association with any of the examined features. Cofactor-free REST sites showed a depletion of both promoter and common REST binding (Fig. 4B). These observations were generally reproduced with data from the Hep G2 cell line (Fig. S6).

We then asked how variable cofactor association played into the transcriptional regulation on REST targets. A previous study [15] has shown that in mouse ESCs, REST-binding sites colocalized with SIN3 or CoREST occurred more frequently in genes whose transcription was directly repressed by REST, as measured by their upregulation upon shRNA knockdown of REST expression. Since we did not have REST knockdown data in any of the cell types studied here, we contrasted REST targets that were bound by cofactors to all REST targets. In contrast to the previous finding [15], we found that REST-bound sites with SIN3 and CoREST were located to genes exhibiting 28 and 4 times higher transcription, respectively, when compared to all REST targets in GM12878 (Fig. 4C; data for Hep G2 in Table S7). This was due to the strong positive association between SIN3 occupancy and active transcription, since REST-SIN3 only targets were expressed 47 times higher than REST targets (Table S8) and SIN3 was the transcription factor most associated with active expression among the 3 cofactors assessed. This result is surprising because SIN3 interacts with HDACs and reduction of histone acetylation is often associated with gene repression, although a previous study has reported that HDACs were localized to many active genes [67]. Moreover, REST sites colocalized with SIN3, CoREST and EZH2 were actually found at genes (e.g., *CHRNB2*) that were expressed 26 times more highly than the median of all REST targets. REST sites colocalized with EZH2 and CoREST, either alone or together, were associated with genes (e.g., *DRD3*, *HTR3A*, and *BDNF*, respectively) expressed at a level of 2.0, 3.6, and 1.9 times lower, respectively, in relation to all REST targets. REST targets without any of the three cofactors were expressed, unexpectedly, at 2.5 times lower levels than all targets in GM12878. The expression difference associated with differential cofactor binding generally held true by examining either all REST peaks or only promoter peaks. In addition, similar results were obtained with data from Hep G2 cells (Table S7), except that in Hep G2 the REST targets not exhibiting colocalization with any of the three cofactors were actually expressed at levels 3.5 times higher than all REST-bound genes. In Hep G2 cells, the REST sites that colocalized only with CoREST were also more highly expressed. The opposite trends observed for REST-alone targets and the REST-CoREST “only” targets between GM12878 and Hep G2 cells suggest that a different set of other REST cofactors may have been recruited to those targets, also in a cell-specific manner. It would be interesting to study in the future if G9A, CTBP, MECP2, LSD1 or other yet-to-be-identified REST interactors are involved. Notably, we did not observe pathways specifically enriched in any group of REST targets with different cofactor occupancies. In summary, our results indicate that SIN3 co-localization was correlated with higher expression while EZH2/CoREST co-occupancy was associated with lower levels of expression of REST targets, with SIN3 seemingly dominant over EZH2/CoREST. In the future, it would be interesting to test experimentally if SIN3 and EZH2/CoREST indeed confer opposite regulatory roles in some REST targets by knocking down these chromatin factors.

The fact that the outcome of REST regulation is largely dependent on its cofactors is not entirely surprising, but it reinforces the view that REST is a molecular platform for recruiting chromatin modifiers, which ultimately determine the transcription activity. To address this computationally, we combined the cofactor colocalization information with histone modification data in GM12878 cells from a previous study [68]. We observed that 82% of the REST-EZH2-only, 74% of the REST-CoREST-only, and 19% of REST-SIN3-only sites were located to regions enriched with H3K27me3 (a repressive histone mark). On the other hand, 74% and 48% of the REST-SIN3-only, 6% and 2% of REST-CoREST-only and 12% and 3% of REST-EZH2-only sites were located to either H3K4me3 (an active histone mark) or H3K27ac (an active histone acetylation mark) enriched regions, respectively. These enrichments are congruent with the known addition of H3K27me3 by EZH2 and removal of H3K4me by LSD1/CoREST. SIN3 acts on the chromatin through the recruitment of HDACs. Since there was no HDAC ChIP-seq data in GM12878 cells, we only studied the colocalization of SIN3 and HDAC2 in Hep G2 cells. A total of 32,895 HDAC2 peaks were called for Hep G2. We found that 69% of the REST sites that colocalized with HDAC2 (n = 374) were also enriched with SIN3 (binomial test, p = 7.5E-129), supporting SIN3-HDAC interaction at REST-bound sites. All together, our study demonstrates that distinct combinations of chromatin modifying cofactors are recruited to different REST-binding regions, and that they likely contribute to the transcriptional outcome of REST regulation. Whether and how these cofactors work together with REST to activate or repress gene expression cannot be directly addressed here, due to the limitation of computational work, and thus requires more study in the future.

### REST binding in neurons exhibits features distinct from REST occupancy in non-neuronal cells

Throughout our analysis the H1-derived neurons stood out from the other cell types. For instance, neuronal REST peaks were enriched with a different motif and REST targets in neurons were overall highly transcribed. This raises the question of whether different cofactors are recruited to the REST-bound sites in neurons. Unfortunately, TAF1 and RNA polymerase II were the only DNA-binding proteins that had been analyzed by ChIP-seq in the same neuron samples, both of which are unlikely to play a deterministic role in REST-specific gene regulation. We have, however, analyzed several available genomic and epigenomic data sets in order to gain a glimpse at the neuron-specific REST function.

As it has been suggested that a REST isoform (*REST4*) could inhibit REST function and activate REST targets [9] and that *REST4* expression is neuron-specific [44], we examined whether this isoform was more abundant in neurons. Our analysis of RNA-seq reads mapped to *REST4* specific exon, however, did not find evidence that *REST4* was the dominant *REST* isoform in neurons. In fact, in comparison to the full length REST (2.7 FPKM), REST4 transcription was 7-fold lower at 0.39 FPKM (Fig. S4). Next, we asked if the presence of small RNAs within REST-binding sites might have altered REST-mediated gene regulation, since it has been shown that the transcription of enhancer RNAs (eRNAs) is often correlated with gene activation [69], [70] and that a double stranded small RNA was shown to activate REST targets [71]. We utilized small RNA sequencing data from the ENCODE project for H1-derived neurons, GM12878, and H1 ESCs [72], and determined small RNA read abundance at individual REST peaks using the algorithm HTSeq [73]. Interestingly, we found in neurons that (i) 804 of the REST sites (15%) had small RNAs mapped to them (≥1 reads per kb per million small RNA-seq reads (RPKM); binomial test, p<2.2E-16), and (ii) genes associated with these REST peaks (n = 761) were expressed at significantly higher levels (median FPKM = 15.0) than all REST bound genes (p = 2.8E-39). In contrast, very few genes were associated with REST peaks that could potentially produce eRNAs by the same criterion in both GM12878 (n = 33) and H1 ESC (n = 69). This difference remained when the threshold for small RNA presence was set to 0.1 RPKM (data not shown).

**Figure 5:**
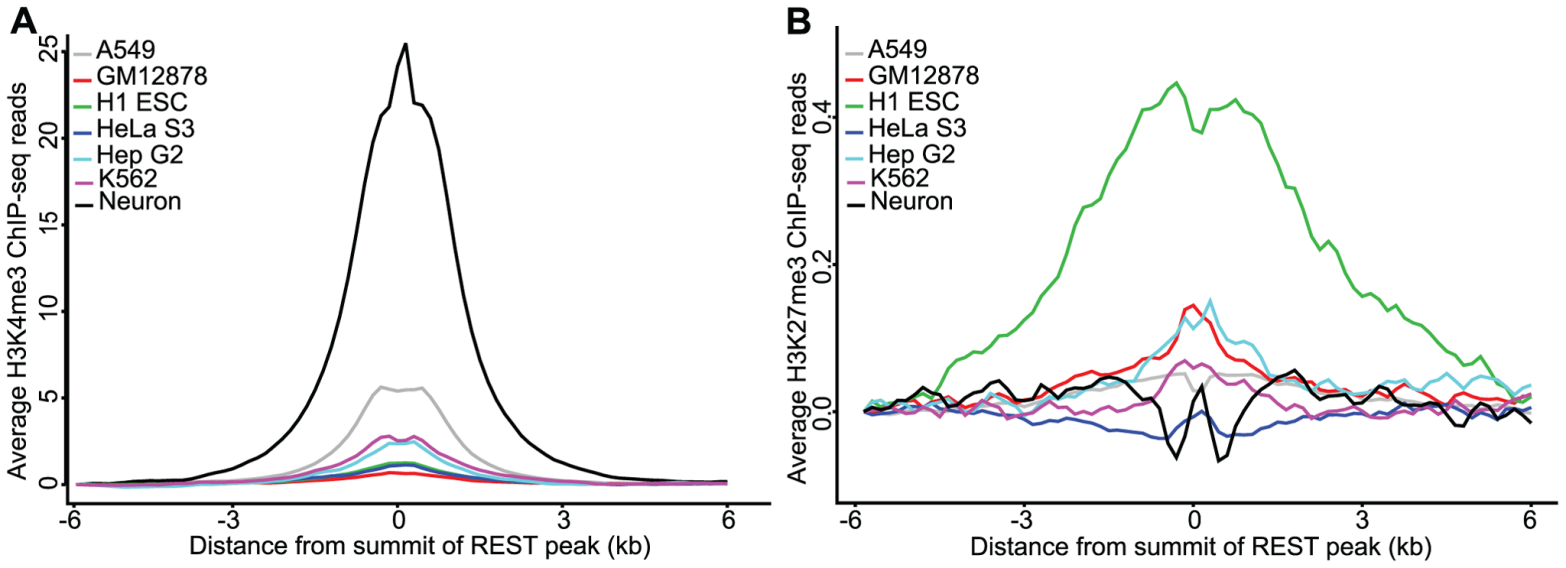
Histone modification profiles at REST peaks. Profiles of (A) H3K4me3 and (B) H3K27me3 ChIP-seq signal at REST peaks in GM12878 cells, H1 ESCs, neurons, A549, HeLa S3, Hep G2, and K562 cells. Y-axis shows the read density from Zhu *et. al.* [68] or ENCODE [34] per 150 bp averaged over REST-bound peaks in each cell type from −6 kb to 6 kb of the peak summits. Data were normalized to a read depth of 5 million mapped reads.

Next, we examined the chromatin modifications at REST binding sites. We obtained chromatin regions that were determined to be enriched with H3K27me3, H3K36me3, H3K4me1, H3K4me3, and H3K9me3 from a previous report, in which these modifications in H9-derived neurons, GM12878 cells, H1 ESCs, and many other tissues were studied [68]. Intersecting those histone modification regions with the REST peaks in neurons, GM12878, and H1 ESCs demonstrated that a much greater percentage (49%) of neuron REST peaks overlapped with H3K4me3, an active histone modification, than did GM12878 (20%) or H1 ESC (18%) REST peaks. This finding was further supported by the high enrichment of H3K4me3 ChIP-seq signal at the center of REST peaks in neurons but not in GM12878 cells, H1 ESCs, A549, HeLa S3, Hep G2, or K562 cells (Fig. 5A). The overlaps of REST peaks with chromatin regions enriched with two additional active histone marks: H3K4me1 and H3K36me3, were also 2× higher in neurons than in either GM12878 or H1 ESCs (Table 2). Conversely, a significantly greater percentage (69%) of GM12878 REST peaks overlapped with H3K27me3 regions, a repressive histone modification, than did neuron REST peaks (27%). This finding was further supported by the enrichment of H3K27me3 ChIP-seq signal at the center of REST peaks in GM12878, Hep G2 and other cell lines, but not in H1-derived neurons (Fig. 5B). Although only 18% of the REST peaks in ESCs overlapped with H3K27me3 regions (Table 2), H3K27me3 ChIP-seq signals showed significant enrichment at REST peaks in ESCs (Fig. 5B), which was upon further examination due to strong H3K27me3 signals in these 18% overlapping peaks (no enriched profile detected after their removal, data not shown). Similarly, a higher overlap of REST peaks with H3K9me3 regions was observed in GM12878 cells compared to either neurons or ESCs. It is possible that lower cofactor colocalization at these neuron REST sites, could explain the higher levels of H3K4me1/3 and lower levels of H3K27me3 and H3K9me3. Likewise, for the 15 known REST target genes, we found much higher association of their REST sites with repressive histone marks outside neuronal cells, 14 of the 15 genes with H3K27me3, and 4 of the 15 with H3K9me3 in GM12878 compared with 6 of the 10 genes with H3K27me3 and none with H3K9me3 in neurons. These differential histone modification colocalizations go hand in hand with the higher expression of these genes in neurons based on RNA-seq data. This finding underscores the hypothesis that distinct sets of REST cofactors are recruited to REST-bound regions in a context-dependent manner, as these 15 genes are occupied by REST across cell types but exhibit differential enrichment of histone marks.

**Table 2:** Overlap of REST peaks with genomic regions enriched with histone modifications.

	% of REST-bound sites			Fold Enrichment over randomly selected genomic regions		
	Neurons	GM12878	H1 ESC	Neurons	GM12878	H1 ESC
**H3K4me3**	49.2%	19.5%	18.3%	19.62	3.44	5.31
**H3K4me1**	29.8%	9.5%	14.8%	4.69	1.78	3.28
**H3K36me3**	45.2%	18.9%	15.5%	1.92	0.88	1.04
**H3K27ac**	N/A	9.6%	6.4%	N/A	3.64	5.34
**H3K27me3**	27.0%	69.2%	17.8%	0.99	1.70	3.17
**H3K9me3**	4.4%	20.8%	2.8%	1.04	1.21	0.67

**Figure 6:**
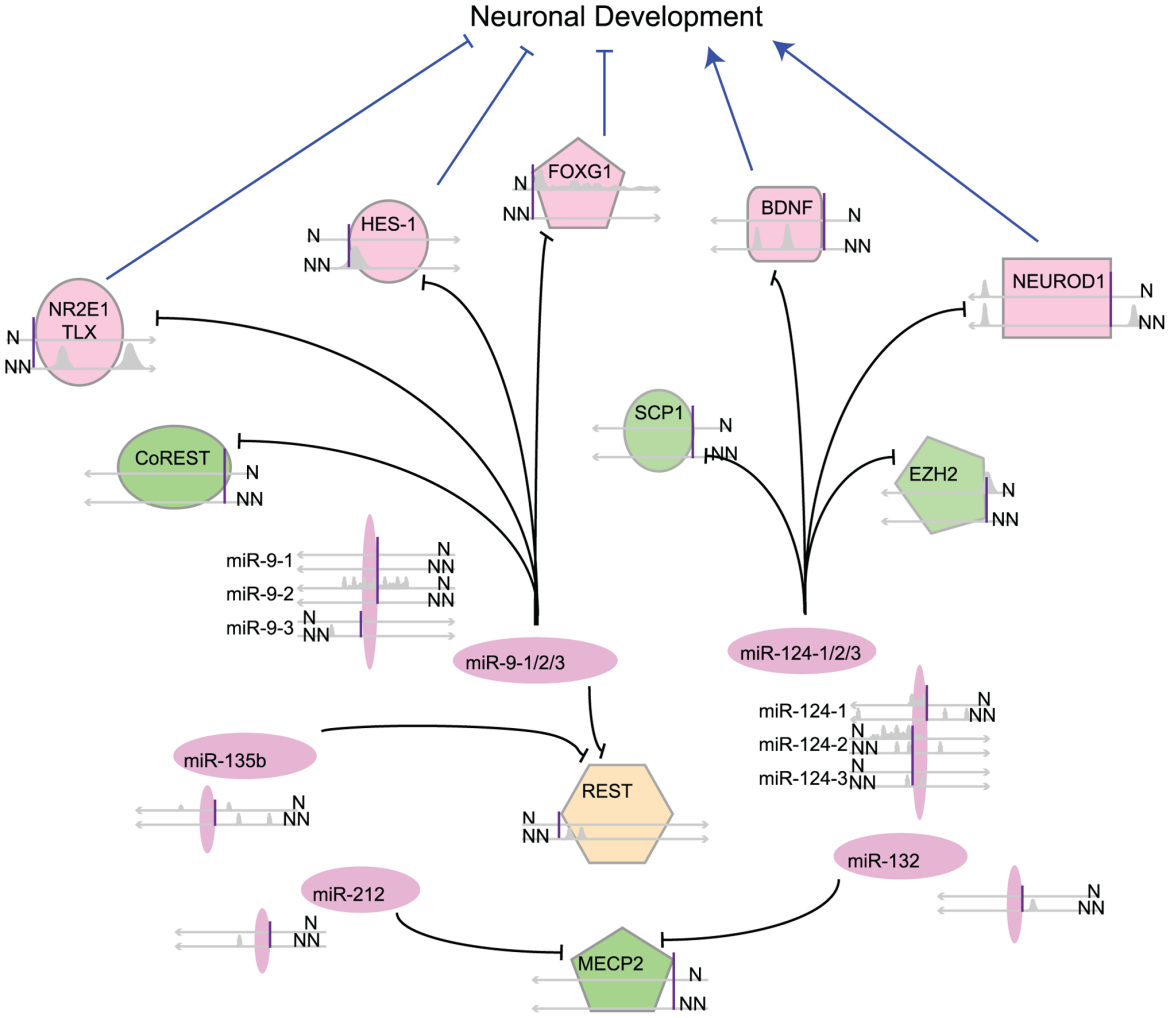
Key neural miRNAs and factors regulated by REST. REST peaks for each gene or miRNA are shown as a cartoon, represented on two lines to indicate REST binding in neurons (N) and non-neuronal cells (NN). TSS is marked by a purple vertical line and direction of transcription indicated by arrows. Black lines indicate miRNA regulation. MiRNAs in purple, REST cofactors in green, transcription factors in pink and their actions on neural development indicated by blue arrows (activate) or flat lines (repress).

We next examined how REST differentially regulated a mini regulatory circuitry that has previously been suggested to be important for neurogenesis. The circuitry includes REST cofactors, several miRNAs, and neurogenic factors critical for neuronal development (Fig. 6). Among the known components of the REST complex [9], [11], [12], [48], *LSD1*, *BRG1*, *HDAC4*, and *HSPC1* (a component of PRC1) were bound by REST in 5 or more cells, frequently including ECC-1, H1 ESCs, HL-60, PANC-1 and SK-N-SH cells. *EZH2* showed REST binding at its promoter in neurons but not other cells (see Table S2).

Finally, we examined the expression of and REST-binding at several miRNAs that have been reported to control neural development [74]–[77]. The expression levels of miRNAs were determined from small RNA-seq datasets. Of the 939 miRNAs annotated in miRBase [38] 134 of them (14%) were bound by REST in at least one of the 16 cell types and 39 of them (4.2%) were differentially bound in neurons and non-neuronal cell types. 32 of the 39 REST-bound miRNAs in neurons (82%) were bound either only in neurons (n = 20) or at a different genomic position in the non-neuronal cells (n = 12). Five miRNAs have been extensively studied for their interaction with REST and their roles in promoting neurogenesis: *miR-9* [9], [78], [79], *miR-124* [9], [79], *miR-132* [9], [79], [80], *miR-135b* [78] and *miR-212* [80], many of which directly target neuronal genes. Interestingly, each of these miRNAs showed a different pattern of REST binding in neurons and non-neuronal cells (Fig. S7), with *miRs 124-3*, and *132/212* targeted by REST in all cell types but neurons, with *miRs 9-3*, *124-1*, *124-2*, and *135b* targeted at a position in neurons different from other cells, and *miR-9-2* bound only in neurons. Compared to their expression in H1 ESCs and GM12878 cells, all of these miRNAs were more highly expressed in neurons (Table 3), suggesting that REST may play a role in activating instead of repressing these miRNAs in neurons. In support of this hypothesis, the expression of *miR-9* was indeed downregulated in mouse neuronal stem cells upon conditional knockdown of REST expression [81].

**Table 3:** MiRNA transcript abundance (RPKMs).

miRNAs	GM12878	H1 ESC	Neurons
***miR-124-1***	0.00	61.23	810.04
***miR-124-2***	2.28	124.63	3,559.19
***miR-124-3***	1.91	147.51	4,576.90
***miR-132***	92.50	5.77	157.87
***miR-135a-1***	0.13	0.84	621.19
***miR-135a-2***	13.75	577.21	6,361.50
***miR-135b***	50.48	116.80	2,157.31
***miR-212***	36.32	0.85	99.08
***miR-9-1***	4,709.96	3,668.15	53,597.92
***miR-9-2***	5,181.70	3,700.43	61,491.03
***miR-9-3***	28.19	24.43	1,176.68

## Discussion

In this study, we have characterized genome-wide REST occupancy across multiple cell types and related that to other transcription factor binding and transcriptional outcome. With REST ChIP-seq data from 16 different human cells, we made an attempt to estimate the number of potential *in vivo* REST binding sites in the human genome. Although we called a total of ∼62,000 REST peaks, they were highly redundant. 63% of the total bindings in the 15 non-neuronal cell types could be identified using data from just three cell types with the most peaks (Fig. S8). Starting from the cell type (H1 ESC) with the most peaks, we computed the number of new REST peaks that were identified by including data from additional cells. As shown in Fig. S8, the number of additional peaks added with each cell line decreases and approaches a plateau at about 7 cell types. The extrapolation of our data indicates that there are ∼18,000 potential REST-bound regions in the human genome. This estimation, however, excludes neurons, because our analysis shows that REST binding in neurons is quite distinct from non-neuronal cell types and much is yet to be learnt for the diversity of REST bindings among neuronal subtypes [7], [30]. We should note that of the genomic sites previously predicted to contain cRE1 motifs [13], almost all (234/235) of the most highly confident ones were included in our REST peaks and additionally 85% (669/783) of those lower confidence peaks in non-repetitive regions were also covered by our REST peaks.

Our findings indicate that the REST cistrome in differentiated human neurons is dramatically different from those in non-neuronal cells. This difference, as well as differences in the dynamics of REST targeting, was also observed in previous studies of REST occupancy at promoters during the development of a variety of mouse neural lineages using microarrays [27], [30], [31]. The limitation of the current study is that the samples used for ChIP assays contained a mixture of neuronal subtypes, which may have differences in REST occupancy. The maturation status of the cultured neurons could be another important factor. From that perspective, the differences between the neuronal REST cistrome and that of non-neuronal cells may actually be larger than what we report here, since the limited overlapping could be due to the presence of a small number of neuronal progenitor cells in the ChIP samples. We should mention that RNA-seq data for our analysis of gene expression in neurons were not derived from the same sample used for ChIP-seq experiment: H1 ESC derived neurons for ChIP and iPSC derived neurons for RNA-seq. The differentiation protocols, however, were essentially the same and promoted the generation of GABAergic and Glutamatergic neurons, and both samples were harvested after four weeks of culture. The enrichment of neurons in our cell culture was supported by the high expression of neuronal and synapses genes [82]: (*TUBB3*, 431 FPKM; *MAP2*, 141 FPKM; *MAPT*, 86 FPKM; *SYN1*,16 FPKM; *DLG4*, 41 FPKM), low expression of genes marking astrocyte and oligodendrocytic glia [82] (*GFAP*, 6 FPKM; *MBP*, 0.5 FPKM; *OLIG2*, 0.7 FPKM), as well as low expression of NSC/NPC markers [83]. We should point out that REST expression in Glutamatergic and GABAergic neurons has been demonstrated previously by co-staining of REST and neuronal specific marks (see Fig. S2 in ref [30]) and recently mRNA and protein expression in neurons in the prefrontal cortex has been reported [84]. In addition, 21% and 25% of REST promoter targets previously found in mouse GABAergic and Glutamatergic neurons [30] were also identified in our neuron ChIP-seq data, respectively. We do not think the sample “mismatch” undermines our finding that REST targets exhibited active expression in neurons but lower expression in non-neuronal cells, because results from our analysis of histone modification ChIP-seq data collected for another neuronal sample (derived from H9-ESC line) also support our conclusion. Nevertheless, it will be important to revisit this issue when the RNA-seq data for H1-derived neurons becomes available from the ENCODE project.

The lack of RE1 motif enrichment in the neuronal REST peaks is quite surprising and intriguing. Although motif analysis identified GGAAA/TA as a potential alternative binding sequence, we do not think it represents true REST recognition motif in neurons, because it is a rather common motif and present in 9% of randomly selected genomic sequences. Instead, a more likely scenario is that REST loses its direct interaction with neuronal chromatin and becomes a co-factor for other transcription factors. Our study showed that small RNAs were enriched in the neuronal REST peaks, but more direct experimental assays are needed to investigate if small RNAs or long non-coding RNAs have a role in altering REST interaction with chromatin and its targets. REST4 has been associated with active gene expression in neurons. Although we did not uncover evidence that REST4 was the dominant isoform in neurons, we did observe that REST4 transcript was increased 3-fold in the transition of day-14 to day-27 neurons (data not shown). Furthermore, our RNA-seq data indicated that *SRRM4*, shown to promote alternative splicing to generate REST4 transcript [85], was transcribed only in neurons, with 14.0 FPKM in neurons but <0.95 FPKM in all non-neuronal samples. This suggests that REST4 could be the dominant REST protein product in our neuron cultures. Nevertheless, REST4-bound chromatin sites would still be expected to be enriched with variants of RE1 motifs. Therefore, it remains a mystery how REST changes its DNA recognition specificity in neurons. Only with further study can we address if REST interaction with the neuronal genome is mediated by other factors, like in the case of recently reported cellular dependent chromatin interaction of TCF7L2 [86]. We need to point out that the lack of RE1 motif enrichment has also been reported in a previous analysis of REST regulation in mouse neurons [30]. We should also emphasize that two ChIP-seq replicates from the H1-derived neurons showed consistent REST binding and the same lab produced all the REST ChIP-seq data with the same antibody, except those from CD4+ T cells. Thus, we do not believe the unique features of neuronal REST cistrome is due to any experimental issues.

Our study sheds new insight into REST regulation of many genes that have critical roles in neuronal development and function (Fig. 6), including *miR-9* and *miR-124* (Fig. S7). These miRNAs contribute to neural differentiation, neural fate determination and cell cycle exit through the repression of a number of neural transcription factors including TLX, FOXG1, HES-1 (all repressed by *miR-9*), REST, and some of its cofactors (Fig. 6). TLX is critical for maintaining neural progenitor cells (NPCs) in their undifferentiated state [87]. HES-1 is required for NSC homeostasis/maintenance [88], as its repression accelerates, while its overexpression inhibits, neurogenesis [89]. FoxG1 maintains NPC self-renewal [90] and suppresses the formation of early-born neurons [91]. Our analysis showed that REST bound to *HES1* and *TLX* in nearly all cell types but neurons, while REST occupied *FOXG1* only in neurons. There is more than one locus encoding miR-9 and miR-124 in the human genome. The neuronal expression of *miR-9* and *miR-124* was likely promoted by the differential promoter REST binding in neurons, as well as certain loci not bound by REST altogether (Fig. S7). Together, these suggest that REST is involved in *miR-9* and *miR-124* transcription and consequently that their repression of neuronal factors that are critical for maintaining the fate of neural stem cells. Simultaneously, REST also directly represses those factors in non-neuronal cells. For two transcription factors that promote neuronal differentiation, *NEUROD1* and *BDNF*, expression is also controlled by REST; both exhibit similar regulation by REST in all of our analyzed cell types except neurons (*NEUROD1* lacks upstream REST binding in both neurons and T cells). Their expression in neurons, however, is also modulated by *miR-124*, likely to strike a balance between factors that activate and inhibit neurogenesis. Several other key neuronal factors are also regulated by the REST/miRNA regulatory circuitry (see Table S2), including BAF53a/b and EFNB1, as well as two (*MYT1L* and *POU3F2*) of the three genes sufficient to convert fibroblasts to neurons [92] and a transcription factor (NeuroD2) that speeds their miRNA-mediated conversion [93]. These results, along with previous findings in the literature, indicate that REST plays an important role in neurogenesis by both directly targeting key neuronal transcription factors and regulating the transcription of neuronal miRNAs. Together, the mini REST/miRNA regulatory network controls neurogenesis synergistically by fine-tuning the expression of individual components to maintain a balance, which is necessary for the proper development of multiple neuronal lineages and for maintaining some level of developmental plasticity.

We were intrigued to discover that REST bound to its own promoter in all 15 cell types but not in neurons. It may be that in neurons, the dynamics of REST production and degradation are different, for example, perhaps more post-translational control is taking place. It is known that REST is a protein with high turnover [10] and that negative auto-regulation speeds response times of transcriptional networks [94]. It could be that less steady-state transcription of REST leads to phases of lower levels of available REST despite the fact that REST expression levels are similar (ranging from 2.6–10.2 FPKM) in neurons and other cell types (Fig. S4). *REST*, itself, along with other members of the REST complex, such as *SCP1*, *EZH2*, CoREST (*RCOR1*) and *MECP2*, are all regulated by REST-bound and brain-specific miRNAs, including *miR-9* and *miR-124*. Many of the REST complex components, miRNAs and REST targets are also regulated by CREB [95], a potential positive regulator of all the REST-regulated miRNAs. Therefore, all of these cofactors are controlled at a number of different levels through REST and its downstream miRNAs. It will be important to study how these interconnected regulatory factors are involved in the formation and function of the REST complexes, especially during neurogenesis, neuronal differentiation and maturation.

## Materials and Methods

### ChIP-seq analysis for CD4+ T cells

The protocols for ChIP-seq and ChIP-qPCR have been described [96]. In brief, human primary CD4+ T cells were purified from blood as previously described [97]. This cell type was chosen because it is an easily accessible primary cell type and known to express REST [3]. Twenty million cells were cross-linked by formaldehyde. Following sonication, chromatin fragments were immunoprecipitated with an anti-REST antibody [Millipore 07-579] and then prepared for sequencing. Sequencing was performed on an Illumina HiSeq 2000 machine. ChIP-seq reads were processed by the Illumina Analyzer Pipeline and aligned to the human genome (GRCh37/hg19) using Bowtie [98]. Unique reads mapped to a single genomic location (allowing up to three mismatches) were kept for peak identification. The primers used for qChIP analysis were listed in Table S9. The ENCODE ChIP-seq data (Table S1), including the one for H1-derived neurons, were collected with a REST antibody provided by Dr. David Anderson at Caltech. Sample information for the H1-derived neurons (including its purity) can be found at (http://genome.cse.ucsc.edu/ENCODE/protocols/cell/human/H1_Neurons_Round1.pdf).

### ChIP-seq peak calling

Peaks were called using the SPP pipeline [35], following the guidelines of controlling Irreproducible Discover Rate (IDR) [99] by the ENCODE project [34], with a relatively strict IDR threshold (0.001). Where multiple ChIP-seq replicates were available, reads from all replicates were combined for peak calling. For T cell data, which did not have a replicate, we divided the ChIP-seq data into two halves to generate pseudoreplicates, and the IDR threshold was calibrated from analysis of the pooled-pseudoreplicated data of the other 15 cell types. We also filtered out REST peaks that mapped to genomic regions red-flagged by the ENCODE (both Duke mappability regions and ENCODE Dac mappability consensus regions) [34], as they were likely a result of experimental artifacts.

### Identification of RE1 motif within REST binding sites

We used MEME 4.6.1 [36] to find enriched motifs within 200 bp sequences centered on the summits of all the REST peaks in the CD4+ T cell data. The resultant RE1 motifs were used to identify peaks with RE1 motifs by the program MAST in MEME suite, in all cases by scanning 200 bp sequences around peak summits and using default parameters.

### Identification of genes with REST binding

The genes containing peaks were identified using in-house scripts. We used RefSeq [37] and miRBase [38] annotations from the UCSC Table Browser (http://genome.ucsc.edu), which provides the precursor forms of miRNAs; in cases of overlapping transcripts from the same gene we picked the longest one for both ChIP-seq and RNA-seq analysis, resulting in 23,010 genes and 928 miRNAs. The script assigned peaks to genes in the following step-wise manner: to promoter regions (−5 kb to +1 kb of TSSs (transcriptional start sites)), to exons, to introns, to distal regulatory regions (−50 kb of transcription starts to +50 kb of transcription ends). When mapping peaks to either promoter regions or distal regions, only the gene with the closest TSS was selected. A single base overlap was used as a rule for these assignments. A peak can be mapped to multiple genes, but only it is equidistant from the TSS of multiple genes, or if it is located to exons or introns shared by multiple genes. Notably, the definition of exonic and intronic REST binding was not applicable to miRNAs and their precursors, as they are short.

### Identification of common peaks and cell-specific peaks

The merged and non-redundant REST peaks were compared to the peaks originally called for each cell type by the SPP program, and those overlapping with peaks from all cells were defined as “common peaks.” For the rest of the merged peaks, we computed a sequencing-depth-normalized maximal read coverage at the 300-bp surrounding the peak summit (averaged from all cells) of each peak in each cell type, and then inferred cell-specific REST binding based on significantly differential read coverage. Each ChIP-seq read was extended to 200 bp for this analysis. For each peak at each cell, we obtained a REST ChIP-enrichment score (E_j_, j = 1,2, …, 16) that was determined from difference in maximal read coverage between REST-ChIP and input samples and normalized by a scaling factor that quantified the genome-wide background noise. The enrichment scores for all peaks were subject to quantile normalization across cell types before used for Z-score statistics. In the end, we defined cell-specific peaks as those having a high Z-score (>3) in one cell type but low Z-scores (<1) in the rest. Further information on this procedure and more details of the methodology development can be found in the Supplemental Methods (Text S1), where we also presented our exploration of several computational schemes to account for different chromatin structure, different immunoprecipitation efficiency/enrichment and other factors in ChIP-seq experiments of different cell types.

### RNA-seq analysis and data processing

All RNA-seq data have been published (Table S5) except the one from neurons, which was collected from 27-day differentiating human neurons derived from induced pluripotent stem cells (iPSCs), which were made from a healthy male. The derivation of the iPSC line (iPSC-2), neuronal differentiation, and RNA-seq sample preparation have been described in previous publications [100], [101]. RNA-seq reads (from polyA+ RNAs) from replicates (when available) were merged and were subsequently aligned to the human genome (version hg19) by TopHat (v2) [60]. Transcripts from Refseq [37] (with micro- and sno-RNAs removed) were used to determine gene expression by the Cuffdiff tool in Cufflinks package (v2) [102], using options for correcting sequence bias and multiple hits, as well as the default geometric mean normalization. All expression comparisons were carried out by the Wilcoxon test and fold changes between groups were based on the medians of FPKMs, unless stated otherwise. Small RNA-seq data were downloaded from GSE24565 [72] and reads were aligned to the human genome by Bowtie (v2) [103], using the options: –local –sensitive-local –score-min G,0,2. We then counted the number of reads at individual REST peaks or miRNAs [38] using HTseq [73], which only uses uniquely mapped reads, with normalization by peak sizes and the number of total aligned reads to yield a RPKM (Reads Per Kb per Million mapped reads) value.

### Heatmap analysis of ChIP-seq data

The ChIP-seq read density was calculated using the program seqMiner [45], which yielded an array that consisted of the maximal number of overlapping ChIP-seq reads (extended to 200-bp) in 300-bp bins from −3.15 kb to +3.15 kb of the REST peak summits. The enrichment of the sequencing-depth-normalized reads over those of input experiments for each cell type was calculated and the enrichment values were subject to both background normalization [104] as described above. This matrix of enrichment values were finally used to generate heatmaps in Figure 2 with the gplots package [105] in R.

### Data access

All data are publicly available and they can be accessed in the Gene Expression Omnibus (see Table S1 and S5).

## Supporting Information

Figure S1 **ChIP-qPCR validation of REST binding in T cells.** (A) ChIP-qPCR enrichment, shown as % of input DNA; error bars are standard deviation from triplicate experiments. *SOX5* is a negative control. (B) Correlation between enrichments in qChIP analysis and peak enrichment scores from the SPP program.
(EPS)

Figure S2 **Top enriched motif identified in ChIP-seq peaks from human neurons.**
(EPS)

Figure S3 **Average distances between summits of overlapping peaks for individual pairs of cell types.** For simplicity, data was computed for peaks that had no more than one overlapping peak in any cell.
(EPS)

Figure S4 **REST and gene expression.** (A) Boxplots showing FPKMs for all genes in each cell type, plotted as log2(FPKM+0.1). (B) Clustering of cell types based on their expression correlations (log2(FPKM+0.1)). (C) Pileup of RNA-seq reads mapping to REST locus as displayed in the IGV browser (heights scaled to number of mapped reads and displayed in a log scale), with FPKMs shown at right. Red box marks the location of the REST4-specific exon (∼50 bp).
(EPS)

Figure S5 **Transcription levels of REST targets bound by REST with different RE1 motifs.** Expression comparison of the genes bound by REST at sites containing cRE1, ncRE1, half-site, or no RE1 motifs. Asterisks mark significant differences (p<0.05). Data plotted as log2(FPKM+0.1).
(EPS)

Figure S6 **Colocalization of REST cofactors in Hep G2 cells.** (A) Venn diagram of colocalization of SIN3, CoREST, and EZH2 at REST peaks. The square constitutes all REST peaks. (B) Context enrichment of REST peaks bound by different cofactors; fold enrichment is compared to all REST peaks.
(EPS)

Figure S7 **Pileup of REST ChIP-seq reads mapping to *miRNA* loci, as displayed in the IGV browser.** From left to right, Mir9-2, Mir9-3, Mir124-1, Mir124-2, Mir124-3, Mir-132, Mir212, and Mir135b.
(EPS)

Figure S8 **Estimated number of genomic REST binding sites.** Y-axis shows the total of non-redundant REST peaks identified as ChIP-seq data from more cell types (x-axis) were used, from cells with more to fewer peaks.
(EPS)

Table S1 **List of accession numbers and total reads of the REST ChIP-seq datasets.**
(XLSX)

Table S2 **List of all REST peaks identified in all cell types, and their relationship to genes.**
(XLSX)

Table S3 **Pathways consistently enriched across cell types.** Lists of the top 10 pathways enriched (p< = 1E-3) in the most cell types, organized by number of cells enriched in. Red and grey background signifies that p-value is below or above the threshold of 1E-3, respectively. Numbers in cells are −log10(p).
(XLSX)

Table S4 **List of top 10 pathways enriched (p< = 1E-3) in neurons, annotated as Table S3.**
(XLSX)

Table S5 **List of accession numbers and summary of RNA-seq datasets.**
(XLSX)

Table S6 **Association between genomic context and REST target expression.** This table contains the median expression values of REST targets with each context (>1 peak per gene, intragenic, c/ncRE1, common) and their respective controls. It also lists Wilcoxon test p-values from their comparison and fold change of median expression without context over with context for each cell type.
(XLSX)

Table S7 **List of the fold-changes of the median expression values for genes associated to each group of peaks with different cofactor colocalizations in comparison to all REST targets.** The analyses were performed for genes with either any peaks or only one promoter peak, using data from GM12878 and Hep G2 cell lines.
(XLSX)

Table S8 **List of median expression values for the group of genes bound by each transcription factor in GM12878 and Hep G2 cell lines.** Data shown are for genes with any peaks or only promoter peaks.
(XLSX)

Table S9 **List of primer sequences used for ChIP-qPCR analysis in T cells.**
(XLSX)

Text S1 **A file describes the details of our methodology development for determining cell-specific REST binding.**
(PDF)
