# Supplementary material for: Comparison of REST Cistromes across Human Cell Types Reveals Common and Context-Specific Functions
Source: PLoS Comput Biol. 2014 Jun 12;10(6):e1003671. doi: 10.1371/journal.pcbi.1003671 (PMC4055426; doi:10.1371/journal.pcbi.1003671)
Supplement: Text S1 — A file describes the details of our methodology development for determining cell-specific REST binding. (PDF) [file pcbi.1003671.s018.pdf]

Comparison of REST cistromes across human cell types reveals common and context-specific functions

Shira Rockowitz [1], Wen-Hui Lien [2], Erika Pedrosa [3], Gang Wei [4], Mingyan Lin [1], Keji Zhao [4], Herbert M. Lachman [1,3,5], Elaine Fuchs [2], Deyou Zheng [1,5,6,*]

1. *Department of Genetics, Albert Einstein College of Medicine, Bronx, NY 10461, U.S.A.*
2. *Howard Hughes Medical Institute, Laboratory of Mammalian Cell Biology & Development, The Rockefeller University, New York, NY 10065, USA*
3. *Department of Psychiatry and Behavioral Sciences, Albert Einstein College of Medicine, Bronx, NY 10461, U.S.A.*
4. *Systems Biology Center, National Heart, Lung, and Blood Institute, National Institute of Health, Bethesda, Maryland 20892, USA*
5. *Department of Neuroscience, Albert Einstein College of Medicine, Bronx, NY 10461, U.S.A.*
6. *The Saul R. Korey Department of Neurology, Albert Einstein College of Medicine, Bronx, NY 10461, U.S.A.*

*To whom correspondence should be addressed.

Tel: +1 718 839 7215;

Fax: +1 718 430 8785;

Email: deyou.zheng@einstein.yu.edu

Supplemental Methods

Identification of common peaks and cell-specific REST peaks

To study the occurrence of a REST binding event (i.e., ChIP-seq peak) across 16 cell types, we first used a strict criterion to call peaks that were "common" to all cell types and then explored more quantitative metrics to determine cell-specificity of the non-common peaks. We intersected the merged and non-redundant REST peaks (n=21,134) from all 16 cell types with the peaks originally called for each cell type by the SPP program [1], and defined those that had a 1-bp overlap with peaks from all cells as "common peaks." Thus, common peaks were REST peaks called in all cell types by SPP.

This approach by simple overlapping, however, would overestimate peaks specific to individual cells if applied to identify cellular specificity or REST binding, because a peak only called in one cell type could have ChIP-seq signals in other cell types but the signals just fell below the SPP thresholds. To overcome this, we compared the ChIP-seq reads at REST peaks in individual cells to identify those peaks that had significantly stronger signals in one cell type than in the rest.

For each of the merged peaks, we computed in each cell type the maximal read coverage within the 300-bp region surrounding the peak summit using seqMiner [2]. This number was normalized to a sequencing depth of 10 million reads, while the peak summit was the average from all cell types. We tested six different schemes (Equation 1-6) for quantifying REST ChIP-seq enrichment (E) at peaks:

1. $E_{ij} = PC_{ij}$

2. $E_{ij} = PC_{ij}/\overline{BC_{\iota}}$

3. $E_{ij} = PC_{ij} - PI_{ij}$

4. $E_{ij} = PC_{ij}/\overline{BC_{\iota}} - PI_{ij}/\overline{BI_{\iota}}$

5. $E_{ij} = (PC_{ij} + 0.16\,)/(PI_{ij} + 0.16)$

6. $E_{ij} = \frac{(PC_{ij}+0.16)}{(PI_{ij}+0.16)} / \frac{(\overline{BC_{\iota J}})}{(\overline{BI_{\iota J}})}$

where PC_{ij} is the normalized read coverage for cell type *i* (i=1,2,…,16) at peak *j* (j=1,2,…, 21134) from the anti-REST ChIP-seq experiments;

where PI_{ij} is the normalized read coverage for cell type *i* (i=1,2,…,16) at peak *j* (j=1,2,…, 21134) from the input ChIP-seq (i.e., control) experiments;

where $\overline{BC_i}$ is the normalized REST-ChIP-seq read coverage for cell type *i* (i=1,2,…,16) averaged across 21134 non-peak genomic regions randomly selected from the human genome, with the distribution of sizes matched to that of REST peaks;

where $\overline{BI_i}$ is the normalized input read coverage for cell type *i* (i=1,2,…,16) averaged across 21134 non-peak genomic regions randomly selected from the human genome, with the distribution of sizes matched to that of REST peaks.

Here, PC_{ij} measured REST ChIP-seq signals at individual cell types, PI_{ij} controlled difference in chromatin structure (e.g., accessibility), whereas $\overline{BC_i}$and $\overline{BI_i}$ controlled for difference in immunoprecipitation and sequencing. The $\overline{BC_i}$and $\overline{BI_i}$ are used because one ChIP-seq run may have higher background signals due to reduced efficiency of immunoprecipitation or poor library preparation, resulting in smaller % of reads located to peaks. As shown in the Table M1 below, we indeed observed a difference in background signals across the 16 ChIP-seq dataset. We also added the minimal non-zero number of reads per 10 million (0.16, observed in A549) to both the numerator and the denominator of the fold change calculation in Equation 5 and 6 in order to deal with 0s in the denominator.

Table M1: Background correction vectors $\overline{BC_i}$and $\overline{BI_i}$, with 95% confidence intervals from 100 iterations.

Cell Type	$\overline{BC_i}$	$\overline{BI_i}$
A549	1.07 ± 0.0014	1.10 ± 0.0010
CD4+ T cell	1.23 ± 0.0013	1.08 ± 0.0012
ECC-1	1.08 ± 0.0010	1.11 ± 0.0010
GM12878	1.16 ± 0.0022	1.39 ± 0.0024
H1 ES	1.14 ± 0.0014	1.15 ± 0.0014
H1-derived neurons	1.06 ± 0.0015	1.08 ± 0.00084
HCT-116	1.13 ± 0.0011	1.15 ± 0.0022
HeLa S3	1.17 ± 0.0014	1.17 ± 0.0012
HepG2	1.22 ± 0.0018	1.22 ± 0.0020
HL-60	1.06 ± 0.0012	1.16 ± 0.0013
K562	1.19 ± 0.0020	1.19 ± 0.0013

MCF-7	1.11 ± 0.0015	1.05 ± 0.0012
PANC1	1.17 ± 0.0013	1.30 ± 0.0020
PFSK1	1.17 ± 0.0019	1.09 ± 0.0017
SK-N-SH	1.15 ± 0.0015	0.99 ± 0.00076
U87	1.19 ± 0.0022	0.97 ± 0.0017

After the enrichment scores (E_{ij}) were further normalized across cell types (see below), we computed Z-score statistics for each peak *j* in cell type *i,*

$$Z_{ij} = \frac{E_{ij} - \bar{\mu}_{E_j}}{\sigma_{E_j}}$$

Then, we defined cell-specific peaks as those that have a Z-score >3 (see below) in one cell type but Z-scores <1 in the rest. Noted that the scripts and sample files for our methods are available at http://dain.aecom.yu.edu/zhenglab2/ePrint/csPeakAnalyzer/.

We next studied how different computational methods could affect the outcomes of REST peaks identified as cell specific. Without experimentally validated cell specific peaks as a gold standard, we randomly sampled cell-specific peaks and studied by visualization whether the cell specific assignment from each computational option reproduced and corroborated the difference present in the raw ChIP-seq data across cell types.

First, we investigated how the different ways (Equation 1-6) of computing enrichment affected our definition of cell specificity peaks. Figure M1 and Table M2 show the results, which indicated that the numbers of peaks called cell specific using different enrichment methodologies were in general similar for most cell types (14 cell types) except neurons and T cells. However, the usage of input is particularly important for some cell lines. Cell specific peaks for H1 ESCs and ECC-1 were reduced by >50% upon consideration of the input data, for example, when the numbers of cell specific peaks produced by equations 1 and 3 were compared. Looking closely into the effects of the different enrichment calculations, we observed that Equation 5 and 6 (versus 3 and 4) would lead to overestimate "cell-specific" peaks for the peaks with very small number of input reads. For example, the equation 5 called 2.3-11X more cell-specific peaks in ECC-1, GM12878, HL-60, and K562 cells than equation 3. Many of these peaks exhibited significant ChIP-seq signals in additional cell types but were identified as cell-specific simply because of low input reads in the specific cell type, for which the cell specific was called. Taken these

results together, we decided to use equation 4 for computing enrichment, i.e, subtraction with background correction, as it considered input data but was least affected by no or few input reads at some peaks.

Figure M1: Percentages of REST peaks in each type identified as cell-specific from different methods of enrichment calculations.

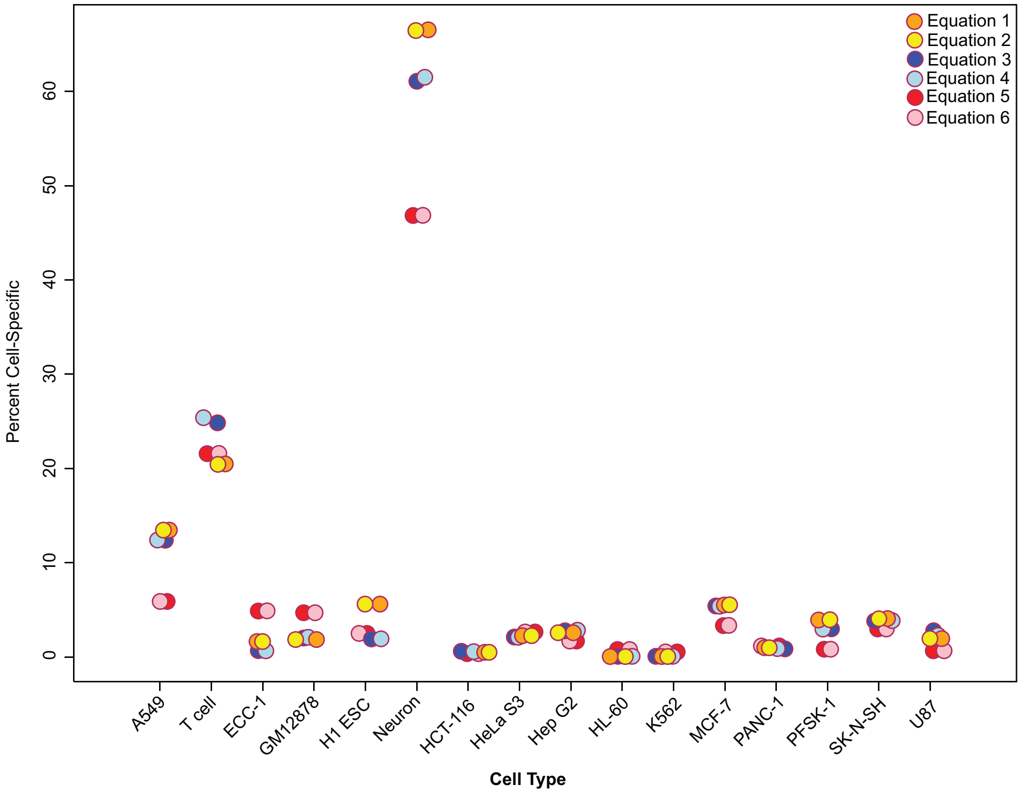

Table M2: Number of REST peaks identified as cell-specific by different ways of enrichment calculations.

Equation	A549	CD4+ T cell	ECC-1	GM12878	H1 ESC	H1-derived neurons	HCT-116	HeLa S3	Hep G2	HL-60	K562	MCF-7	PANC-1	PFSK-1	SK-N-SH	U87
1	626	902	85	47	460	3584	14	93	76	2	3	217	32	138	166	47
2	626	900	85	47	460	3580	14	93	76	2	3	218	32	139	165	47
3	576	1094	35	51	157	3290	17	87	82	3	4	213	28	105	155	67
4	577	1118	34	53	158	3313	16	86	84	3	4	212	29	104	157	55
5	274	950	253	119	205	2523	10	109	50	33	30	131	38	29	121	16
6	274	951	254	119	205	2524	10	109	50	33	30	132	38	29	121	16

Next, we looked into the effect of data normalization across samples. We focused on two normalization methods: quantile normalization, utilizing the quantile normalization function from the R package preprocessCore [3], upper-quartile normalization, using a scaling factor calculation similar to that from edgeR [4]. We studied upper-quartile normalization because it has been recently recommended as a preferred method for RNA-seq data analysis and the numbers of REST peaks called for our 16 samples differed drastically, ranging from 2,408 to 8,199. A comparison of the numbers of peaks called cell-specific using the two normalization methodologies are in Figure M2 and Table M3, as well as the numbers without normalization. Interestingly, for the majority of the cell types, there was not a great difference between the two normalization strategies, but quantile normalization produced fewer HCT-116 and Hep G2 specific peaks that showed signal enrichment in other cell types. Moreover, upper quartile normalization led to significant under estimation of cell specific peaks for the cell types that indeed had quite a large fraction of cell specific peaks, e.g., neurons and T cells. We also investigated geometric mean normalization, using the relative log expression normalization method from edgeR [4], as well as upper quartile normalization scaled to the common peaks found in all 16 cell types. Their performances were quite similar to quantile normalization (data not shown). Furthermore, we have also analyzed how the normalization methods could potentially alter the enrichment scores of the regions without peaks in each cell type (but bound by REST in at least one of the other 15 cell types). As these are non-peak regions, a good normalization methodology should yield a similar distribution of the enrichment scores across samples. Indeed, we found that quantile normalization met this criterion. The distributions of the enrichment scores for these non-peak regions for different cell types were more consistent after quantile normalization in comparison to either before normalization of by upper quartile normalization (Figure M3). Taking together, these results indicated that quantile normalization was appropriate for the comparison of our ChIP-seq data. Note that in our above comparison of different ways to compute enrichment scores, we used quantile normalization.

Figure M2: Percentages of REST peaks in each type that were identified as cell-specific from different normalization methods.

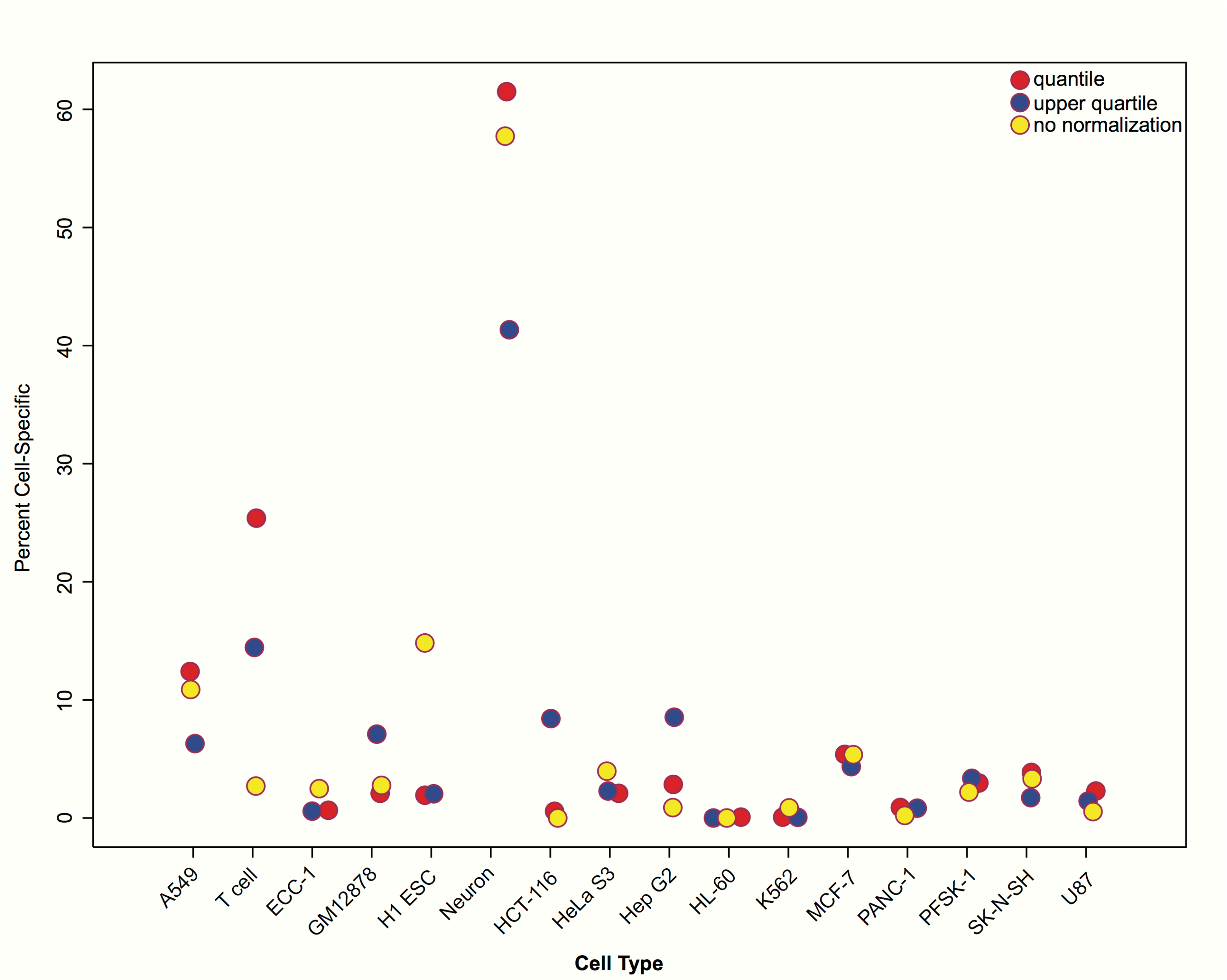

Table M3: Number of REST peaks identified as cell-specific for each cell types from different normalization methods.

	A549	CD4+ T cell	ECC-1	GM12878	H1 ESC	H1-derived neurons	HCT-116	HeLa S3	Hep G2	HL-60	K562	MCF-7	PANC-1	PFSK-1	SK-N-SH	U87
quantile	577	1118	34	53	158	3313	16	86	84	3	4	212	29	104	157	55
upper quartile	293	636	30	180	168	2227	237	94	252	0	3	171	27	118	70	33
no normalization	506	119	129	70	1215	3110	0	163	26	0	48	211	7	77	135	13

Figure M3: Boxplots of enrichment scores of non-peak regions before and after normalization.

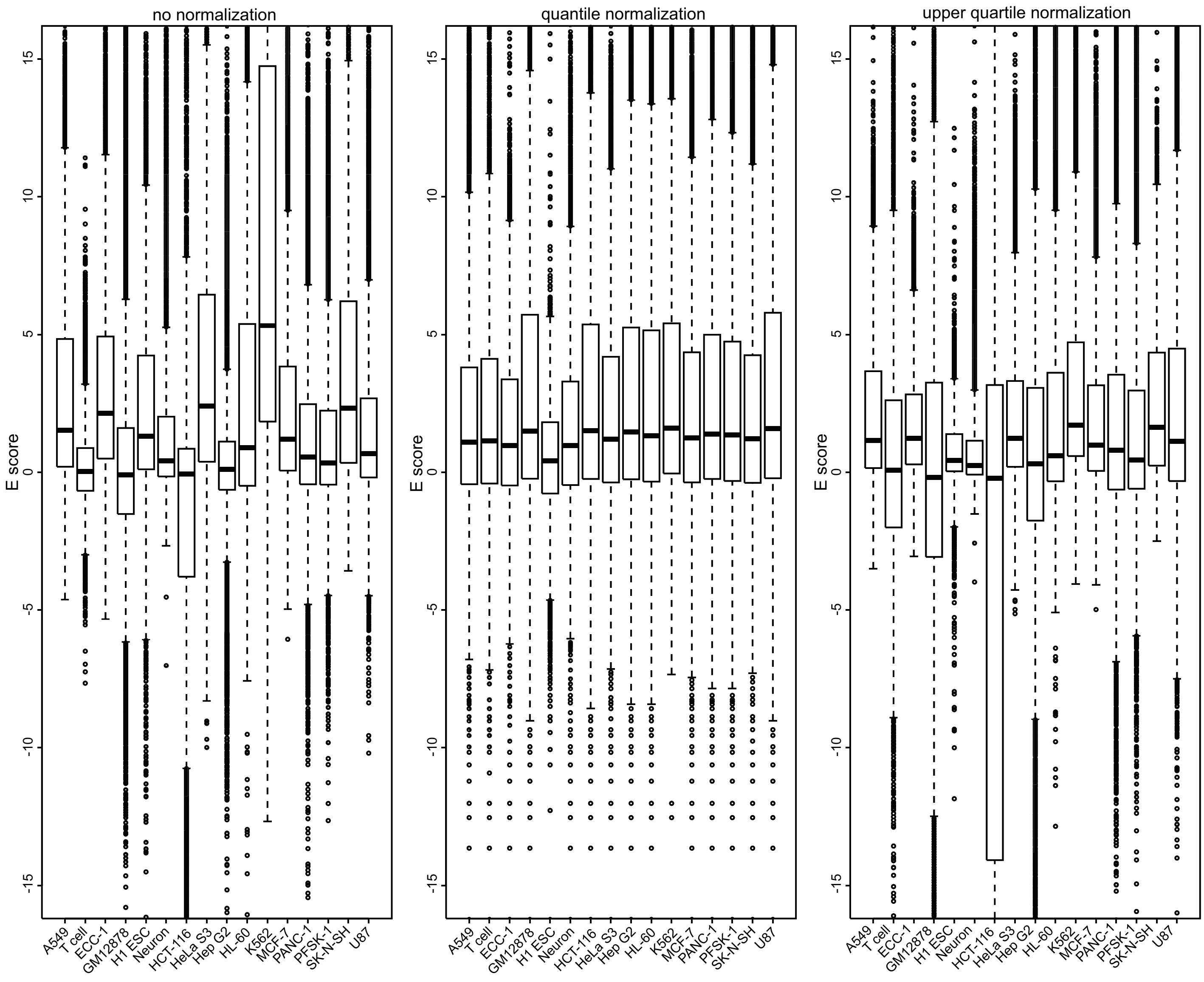

Finally, we examined how Z-score cutoffs might lead to different estimate of cell specific peaks. We tried cutoffs of 2, 3 and 4 and found no much difference between 2 and 3. There were practically not any cell specific peaks identified by the cutoff of 4, as shown in Figure M4 and Table M4, indicating that 4 was too strict. Note that the Z score cutoffs were set to 3 in the above comparison of different computation methods.

In the end, we chose equation 4, quantile normalization, and Z-score cutoff of >3 for our final calling cell specific peaks. We should point out that data from some cell types, e.g., T cell, H1 ESC and H1

neurons, were relatively more sensitive to our choice of methods. The number of REST binding events and the separation of called REST peaks from uncalled binding might be relevant to the difference.

Figure M4: Percentages of peaks identified as cell-specific at different Z-score cutoffs.

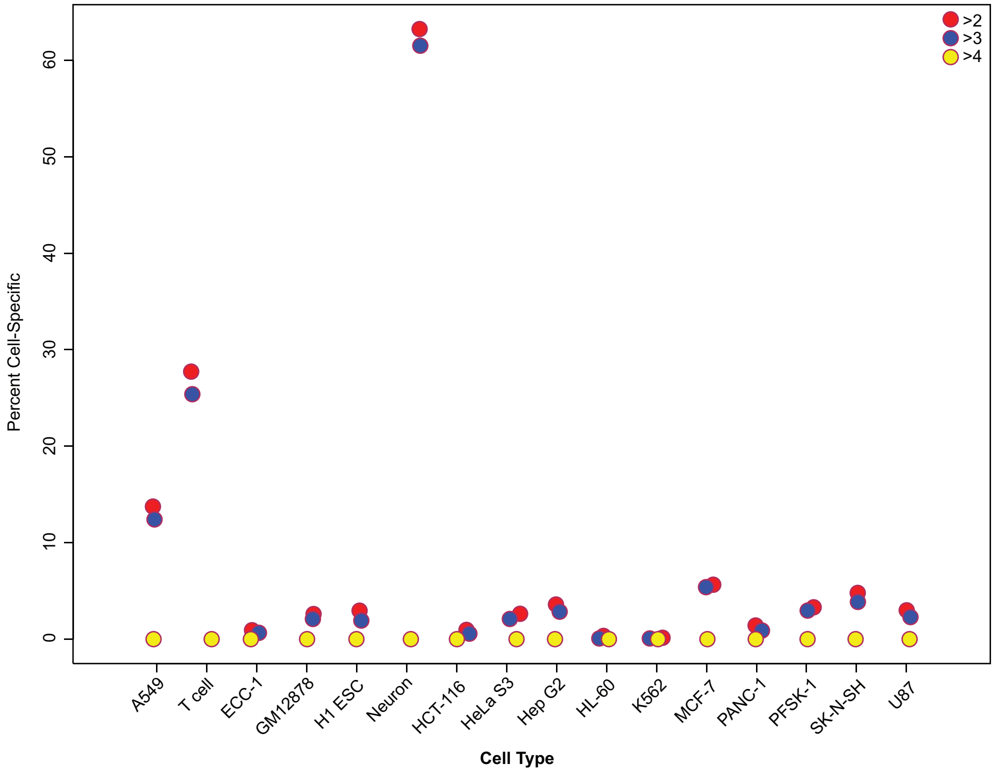

Table M4: Number of peaks identified as cell-specific at different Z-score cutoffs.

Z	A549	CD4+ T cell	ECC-1	GM12878	H1 ESC	H1-derived neurons	HCT-116	HeLa S3	Hep G2	HL-60	K562	MCF-7	PANC-1	PFSK-1	SK-N-SH	U87
>2	639	1221	48	66	242	3406	27	108	106	14	8	222	46	116	195	72
>3	577	1118	34	53	158	3313	16	86	84	3	4	212	29	104	157	55
>4	0	0	0	0	0	0	0	0	0	0	0	0	0	0	0	0

Supplemental References:

1. Kharchenko PV, Tolstorukov MY, Park PJ (2008) Design and analysis of ChIP-seq experiments for DNA-binding proteins. Nat Biotechnol 26: 1351–1359. doi:10.1038/nbt.1508.

2. Ye T, Krebs AR, Choukrallah M-A, Keime C, Plewniak F, et al. (2011) seqMINER: an integrated ChIP-seq data interpretation platform. Nucleic Acids Res 39: e35–e35. doi:10.1093/nar/gkq1287.

3. Bolstad BM (n.d.) preprocessCore: A collection of pre-processing functions.

4. Robinson MD, McCarthy DJ, Smyth GK (2010) edgeR: a Bioconductor package for differential expression analysis of digital gene expression data. Bioinformatics 26: 139–140. doi:10.1093/bioinformatics/btp616.
